# miR-665 expression predicts poor survival and promotes tumor metastasis by targeting NR4A3 in breast cancer

**DOI:** 10.1038/s41419-019-1705-z

**Published:** 2019-06-17

**Authors:** Xin-Ge Zhao, Jing-Ye Hu, Jun Tang, Wei Yi, Mei-Yin Zhang, Rong Deng, Shi-Juan Mai, Nuo-Qing Weng, Rui-Qi Wang, Ji Liu, Hui-Zhong Zhang, Jie-Hua He, Hui-Yun Wang

**Affiliations:** 1 State Key Laboratory of Oncology in South China, Collaborative Innovation Center for Cancer Medicine, Sun Yat-Sen University Cancer Center, Guangzhou, 510060 China; 2 Department of Basic Medicine, Guiyang College of Traditional Chinese Medicine, Guiyang, 550002 China; 3 Department of Breast Cancer, Sun Yat-Sen University Cancer Center, Guangzhou, 510060 China; 4 Department of Radiation Oncology, The First Affiliated Hospital of Guangzhou Medical University, Guangzhou, 510060 China; 5 Department of Breast Cancer, Sun Yat-Sen University Cancer Center, Guangzhou, 510060 China

**Keywords:** Prognostic markers, Breast cancer, Translational research

## Abstract

Cancer metastasis is the main cause of death in breast cancer (BC) patients. Therefore, prediction and treatment of metastasis is critical for enhancing the survival of BC patients. In this study, we aimed to identify biomarkers that can predict metastasis of BC and elucidate the underlying mechanism of the functional involvement of such markers in metastasis. miRNA expression profile was analyzed using a custom microarray system in 422 BC tissues. The relationship between the upregulated miR-665, metastasis and survival of BC was analyzed and verified in another set of 161 BC samples. The biological function of miR-665 in BC carcinogenesis was explored with in vitro and in vivo methods. The target gene of miR-665 and its signaling cascade were also analyzed. There are 399 differentially expressed miRNAs between BC and noncancerous tissues, of which miR-665 is the most upregulated miRNA in the BC tissues compared with non-tumor breast tissues (*P* < 0.001). The expression of miR-665 predicts metastasis and poor survival in 422 BC patients, which is verified in another 161 BC patients and 2323 BC cases from online databases. Ectopic miR-665 expression promotes epithelial–mesenchymal transition (EMT), proliferation, migration and invasion of BC cells, and increases tumor growth and metastasis of BC in mice. Bioinformatics, luciferase assay and other methods showed that nuclear receptor subfamily 4 group A member 3 (NR4A3) is a target of miR-665 in BC. Mechanistically, we demonstrated that miR-665 promotes EMT, invasion and metastasis of BC via inhibiting NR4A3 to activate MAPK/ERK kinase (MEK) signaling pathway. Our study demonstrates that miR-665 upregulation is associated with metastasis and poor survival in BC patients, and mechanistically, miR-665 enhances progression of BC via NR4A3/MEK signaling pathway. This study provides a new potential prognostic biomarker and therapeutic target for BC patients.

## Introduction

Breast cancer (BC) is the second most common cancer in the world and is the fifth leading cause of cancer death among women^1^. A recent report shows that BC ranked first among young adults aged 20–39 years in terms of new cases and deaths in the world^2^. In 2017, approximately 252,710 new cases of female breast cancer are expected to be diagnosed in the United States and about 40,610 women will die from this disease^3^. Importantly, it is not the local tumor itself but the result of metastasis to the lymph nodes or other organs that leads to death. Therefore, prediction, prevention and treatment of BC metastasis are critically important to enhance survival of BC patients^4,5^. To this purpose, a better understanding of the molecular mechanisms underlying the metastasis of BC is desperately needed and will facilitate the identification of novel biomarkers for prediction and therapeutic targets for treatment of metastasis in BC patients.

MicroRNAs (miRNAs) are a class of noncoding RNAs of about 22 nucleotides in length that negatively regulate posttranscriptional gene expression by either repressing the targeted mRNA translation or degrading the mRNAs via partly binding to mRNA’s 3′ untranslated region (UTR)^6^. MiRNAs are now widely recognized to regulate diverse cellular processes in both normal and tumor cells. Studies also show that the abnormally expressed miRNAs are involved in breast tumorigenesis and metastasis by acting as a tumor suppressor or tumor activator^7–10^. However, the role and mechanism of miRNA in BC still have not been fully elucidated^11–13^.

To investigate the role of miRNA in BC development and progression, we examined miRNA expression profile in 422 BC tissues by using a custom microarray system and found that miR-665 was upregulated in BC tissues and associated with poor survival of BC patients. Previous research also reported upregulation of miR-665 in hepatocellular carcinoma, non-small cell lung cancer and intestinal gastric adenocarcinoma^14–16^ and downregulation in gastric signet ring cell carcinoma, osteosarcoma, and pancreatic cancer^16–18^. The role of miR-665 in BC, however, has not been explored yet although one report mentioned miR-665 downregulation in 48 BC tissues^19^.

In the present study, we demonstrate that miR-665 is significantly upregulated in BC and markedly correlated with tumor distant metastasis and poor prognosis of BC. Furthermore, miR-665 acts as a potent oncomiR that promotes cell proliferation, invasion, and metastasis in vitro and in vivo by targeting NR4A3.

## Materials and methods

### Patients and samples

In this study, 422 archived formalin fixed paraffin-embedded (FFPE) BC samples and 31 FFPE non-cancerous breast tissues (NBT) were obtained from the Department of Pathology, Sun Yat-Sen University Cancer Center (SYSUCC), Guangzhou, China. The 422 BC patients with breast cancer underwent curative mastectomy and were pathologically diagnosed as invasive ductal carcinoma (IDC) between June of 1999 and March of 2005. Another 161 FFPE BC samples from patients who received curative mastectomy and pathologically diagnosed as IDC between June of 2002 and December of 2006, were collected from the First Affiliated Hospital of Guangzhou Medical University (FAHGMU), Guangzhou, China. Tumor node metastasis (TNM) staging was performed according to the American Joint Committee on Cancer staging manual (sixth edition, 2002). The clinical characteristics of patients in the 2 cohorts are summarized in Table 1. The median follow-up time was 94.3 months (range from 3 to 142) for 422 patients from SYSUCC and 75.2 months (range from 3 to 116) for 161 patients from FAHGMU. Overall survival (OS) was determined from the date of surgery to the date of death due to any cause or the last follow-up date; disease-free survival (DFS) from the date of surgery to the date of the first distant metastasis, relapse, death or the last follow-up date; distance metastasis-free survival (DMFS) from the date of surgery to the date of the first distant metastasis or death. This study was approved by the Ethical Committees of Sun Yat-Sen University Cancer Center and Affiliated Hospital of Guangzhou Medical University. The written informed consent was obtained from every patient in the two cohorts.

**Table 3:** Cox regression analysis of characteristics associated with survival of BC patients in SYSUCC cohort

Clinical characteristics	Univariate Cox regression analysis		Multivariate Cox regression analysis	
	HR (95% CI)	*P* Value	HR (95% CI)	*P* Value
*Overall survival*				
miR-665 (high vs. low level)	1.818 (1.225-2.696)	**0.003**	1.837 (1.240–2.719)	**0.002**
Age (≥50 vs. <50)	0.998 (0.957–1.041)	0.924		
Menopause (yes vs. no)	0.779 (0.310–1.958)	0.595		
Pathological Grade (III vs. I and II)	1.079 (0.501–2.323)	0.845		
Her-2 (positive vs. negative)	1.084 (0.699–1.681)	0.717		
PR (positive vs. negative)	0.708 (0.451–1.111)	0.133		
ER (positive vs. negative)	0.703 (0.456–1.082)	0.109		
TNBC (positive vs. negative)	0.968 (0.531–1.765)	0.916		
TNM stage (III vs. II vs. I)	2.287 (1.660–3.151)	**<0.001**	2.392 (1.240–2.719)	**<0.001**
*Disease-free survival*				
miR-665 (high vs. low level)	1.618 (1.137–2.303)	**0.008**	1.629 (1.147–2.313)	**0.006**
Age (≥50 vs. <50)	0.916 (0.962–1.035)	0.998		
Menopause (yes vs. no)	0.701 (0.316–1.555)	0.383		
Pathological Grade (III vs. I and II)	1.215 (0.629–2.346)	0.562		
Her-2 (positive vs. negative)	1.068 (0.723–1.578)	0.74		
PR (positive vs. negative)	0.729 (0.481–1.103)	0.134		
ER (positive vs. negative)	0.697 (0.483–1.004)	0.053		
TNBC (positive vs. negative)	1.331 (0.877–2.020)	0.179		
TNM stage (III vs. II vs. I)	2.098 (1.576–2.793)	**<0.001**	2.116 (1.598–2.803)	**<0.001**

The bold values are less than 0.05, which have statistic significance

### Microarray assay

The custom miRNA microarray employed in this study had been fabricated in house and hybridized with total RNAs as previously described^20,21^. Briefly, 1849 probes had been successfully designed based on all human mature miRNAs (1921) in the miRBase database (Release 18.0) according to the principle proposed by Wang et al.^22^. All probes (20–22 nt, 40 μM final concentration) mixed with printing buffer were printed on cleaned slides, and the microarray was hybridized at 45 °C for 16 h with 2 μg total RNA labeled with Cy3 or Cy5. After washing three times, microarray was dried and scanned. After subtracting background, the microarray data were normalized with the quantile normalization method and log transformed.

### Cell culture and transfection

A normal human breast epithelial cell line, MCF-10A and six human breast cancer cells T47D, MCF-7, MDA-MB-231, MDA-MB-415, ZR-75-1, and ZR-75-30 cell lines were maintained at the State Key Laboratory of Oncology in South China. MCF-10A cells were cultured in a complete growth medium (CM-0525, Procell Life Science & Technology Company, Wuhan, China). BC cells were maintained in Dulbecco’s modified Eagle medium (DMEM containing 4.5 g/L d-glucose) supplemented with 10% fetal bovine serum (FBS; Gibco, US) at 37 °C in a humidified incubator with an atmosphere of 5% CO_2_. The miR-665 mimics, miR-665 inhibitor, normal (scramble) control (NC) oligonucleotides, and the small interfering RNA (siRNA) targeting human NR4A3 mRNA were purchased from GenePharma (Shanghai, China). Cell transfection was performed using Lipofectamine™ 3000 transfection reagent (Invitrogen, USA) according to the manufacturer’s instructions.

### Lentivirus production and transduction

The recombinant lentiviral vectors containing miRNA-665, short hairpin RNA against human miRNA-665 or scrambled control oligonucleotides were purchased from GenePharma (Shanghai, China). MCF-7, MDA-MB-231 and ZR-75-30 cells were infected with recombinant lentivirus plus 5 µg/ml polybrene (Sigma, St. Louis, MO, USA) according to the manufacturer’s instructions. Stable transfected cells were selected by using puromycin (1 μg/ml) for 2 weeks. Green flourescent protein-positive cells were collected and used for subsequent assays.

### Quantitative real-time PCR (qRT-PCR)

Paraffin-embedded breast cancer tissues and noncancerous breast tissues were cut into 8–10 µm sections, which were then scraped off the slides and collected into a tube with a modified needle. The paraffin-embedded debris tissues were deparaffinized thrice with xylene. Total RNA was extracted from the debris tissues using the phenol/chloroform extraction method as described previously^20,23^ and extracted from cultured cells or fresh tissues using the TRIzol (Invitrogen, USA) according to the manufacturer’s instruction. For the gene-expression assay, 1 μg of total RNA was used to synthesize cDNA in a 20 μL total volume using a PrimeScript RT reagent kit (Promega, Madison, WI, USA) and 0.4 μL of cDNA product was used for quantitative PCR with the Platinum SYBR Green qPCR SuperMix-UDG kit. For miRNA quantitative real-time polymerase chain reaction (qRT-PCR), 10 ng of total RNA isolated from FFPE samples were analyzed by the TaqMan method; or 1 μg of total RNA extracted from cell lines and miR-665 primers or U6 primers were used to reverse transcribe with PrimeScript RT reagent kit in 20 μL of total volume, and 0.4 μL of the RT products was applied to quantitative PCR with Bulge-Loop™ miRNA qRT-PCR reagent kit (RIBOBIO, Guangzhou, China). The PCR reaction cycle was as follows: 50 °C for 2 min and then 95 °C for 10 min followed by 35 or 40 cycles of 95 °C for 15 s and 60 °C for 60 s. Each sample was analyzed in triplicate. Glyceraldehyde-3-phosphate dehydrogenase (GAPDH) was used as an internal control to calculate the relative gene expression and the universal small nuclear RNA (snRNA) U6 was used as an endogenous control to calculate the relative miR-665 expression. miRNA or gene expression level measured by qRT-PCR was presented as 2^−ΔΔCT^. The primers for miR-665, control U6, and other genes are listed in Table S1.

### Western blot analysis

Cultured cells were harvested and lysed in RIPA buffer containing phenylmethylsulfonyl fluoride (PMSF) (100:1), protease inhibitor (100:1) and phosphatase inhibitor (1000:1) for 30 min on ice. Then the lysed solution was centrifuged at 12,000 *g*, 4 °C for 15 min. Protein in the supernatant was collected and its concentration was measured using a Bicinchoninic Acid Assay kit (BOSTER, Pleasanton, CA). Approximately 10–20 μg of protein were denatured at 100 °C for 10 min with DualColor Protein Loading Buffer (Life, USA). The denatured proteins were separated in 10% sodium dodecyl sulfate-polyacrylamide gel electrophoresis. After electrophoresis, proteins were transferred onto polyvinylidene difluoride membranes (GE Healthcare Life Sciences, UK) using a Bio-Rad Mini-Trans-Blot apparatus at 100 V for 2 h as suggested by the manufacturer. The membranes were blocked in 5% nonfat milk for 2 h followed by incubation with primary monoclonal antibodies overnight at 4 °C with soft shaking. After 3 washes with Tris-buffered saline and Tween 20, the membranes were subsequently incubated with the corresponding secondary antibody coupled to horseradish peroxidase (HRP) at room temperature for 2 h and developed in electrochemiluminescence detection reagent (Advansta, Wuhan, China). The primary antibodies against human E-cadherin, N-cadherin, Vimentin, β-catenin, Slug, extracellular signal-regulated kinase (ERK), p-ERK, and GAPDH were purchased from Cell Signal Technology (Boston, USA). NR4A3 antibody was purchased from Santa Cruz Biotechnology (Dallas, USA).

### Immunofluorescence assay

Immunofluorescence assay was performed 48 h after MCF-7 cells were transfected with miR-665 mimics or NC. The BC cells first were fixed with 4% paraformaldehyde for 15 min and washed 3 times with PBS, and then permeabilized with 0.1% Triton X-100 for 10 min. After washing 3 times with PBS, the cells were blocked with 10% FBS for 1 h. Then the cells were incubated with antibodies against E-cadherin, Vimentin, and β-catenin (Cell Signaling Technology) at 4 °C overnight, respectively. The second antibody (goat anti-rabbit IgG) conjugated with Alexa Fluor 555 (Cell Signaling Technology, USA) was added and incubated at 37 °C for 1 h. After three washes with PBS, nuclei was co-stained with DAPI (Beyotime, Shanghai, China) for 15 min. Finally, cell images were captured using a laser scanning confocal microscopy (Olympus, Japan).

### Cell proliferation assay

Cell proliferation assays were performed using a Cell counting kit-8 (CCK-8, Dojindo Laboratory, Kyushu, Japan) according to the manufacturer’s instruction. Briefly, cells were seeded in 96-well plates at 2.0 × 10^3^ cells/well and cultured for 1–7 days. At the indicated times, 10 µL of CCK-8 solution was added to each well and incubated for additional 2 h at 37 °C. Then the absorbance of the mixed solutions was measured at 450 nm with a SpectraMax M5 Multi-Mode Microplate Reader (Molecular Devices LLC, Sunnyvale, CA, USA). A calibration curve was prepared using absorbance obtained from wells that contained known numbers of viable cells.

### Colony formation assays

Cells were seeded in 6-well plates (500 cells/well) and incubated for 14 days. Cells were fixed with methanol for 10 min and stained with 0.1% crystal violet (Weijia Biology Science and Technology Co., Guangzhou, China) for 30 min. Colonies containing more than 50 cells were counted under a microscope.

### Cell cycle analysis

Cell cycle distribution was examined by flow cytometry using a cell cycle assay kit (BD Biosciences, San Jose, CA, USA) according to the manufacturer’s protocol. After propidium iodide (PI) staining, the cellular DNA content was used to determine cell cycle profile (G0/G1, G2/M and S phases), and the distribution of cell cycle was analyzed using Cell-FIT software (BD Biosciences, San Jose, CA, USA).

### Cell apoptosis analysis

Cells were harvested and washed with ice-cold PBS thrice. Then, the cells were stained with Annexin V-fluorescein isothiocyanate and PI according to the manufacturer’s instruction (BD Biosciences, San Jose, CA, USA) and examined with flow cytometry. The apoptotic cells were determined using Cell-FIT software (BD Biosciences, San Jose, CA, USA).

### Cell migration, wound healing, and invasion assays

For migration assays, cells (5 × 10^5^) in 200 µL of serum-free DMEM medium were seeded in the upper chamber (BD Biosciences) with 8-μm pore membrane of an insert and placed in the 24-well culture plate. DMEM medium (700 µL) containing 10% FBS was added to the lower chamber as a chemoattractant. After 16 h, the nonmigratory cells remaining in the upper chamber were gently removed with a cotton swab. The migrated cells located on the lower side of the insert were fixed with methanol for 10 min and stained with crystal violet (Weijia Biology Science and Technology Co., Guangzhou, China) for 30 min at room temperature. Five random fields per well were observed, and cells were counted under the microscope. In addition, cell migration was also assessed with a scratch wound healing assay. The cells were seeded in a six-well plate. When the cells reached subconfluence, a scratch wound was generated with a sterile micropipette tip, and the 10% FBS medium was replaced with serum-free DMEM. The scratch width was observed every 24 h and photographed under a microscope and the results were presented as percent scratch closure.

For invasion assays, cells (5 × 10^5^) in 200 µL of serum-free DMEM medium were seeded in the chamber (BD Biosciences) with matrigel-coated 8-μm pore membrane of an insert and the insert was positioned in a 24-well culture plate. The subsequent steps were carried out as the same as the migration assay protocol.

### Luciferase reporter assay

To confirm the direct regulating relationship between miR-665 and NR4A3, the putative miR-665 recognition element wild type and mutant sequences in the 3′ UTR of NR4A3 gene was cloned into a SV40-firefly luciferase reporter vector (Applied Genechem., Shanghai, China) for NR4A3. For luciferase reporter assay, the constructed report vectors containing wild-type fragment or mutant-type fragment together with renilla vector and miR-665 mimics or miR-NC were co-transfect into MCF-7 or MDA-MB-231 cells using Lipofectamine^TM^ 3000 reagent. The transfected cells were incubated for 48 h and prepared for measuring the luminescence signals according to instruction of Dual-Luciferase Reporter Assay System kit (Promega, Madison, WI, USA). The firefly luciferase activity was normalized based on Renilla luciferase activity.

To demonstrate that overexpressed miR-665 induces transcriptional activation of β-catenin, the promoter of β-catenin was cloned into the Gaussia Luciferase (Gluc) reporter vector (Genecopoeia, Rockville, MD, USA), a dual-reporter system consisting of secreted GLuc and Alkaline Phosphatase (SEAP). Then the dual-reporter system and miR-665 NC or miR-665 mimics, were co-transfected into MCF-7 or MDA-MB-231 cells with Lipofectamine^TM^ 3000 reagent. Luciferase activity was detected with Secrete-Pair^TM^ Dual Luminescence Assay Kit (Genecopoeia) according to the manufacturer’s instructions. The SEAP signal was used as a transfection efficiency internal control and luminescence intensities were detected by Tecan Spark^TM^ 10 M.

### Tumor formation and metastasis assays in nude mice

Tumor formation assay: 16 female BALB/c nude mice (4–5 week old) were injected subcutaneously with 1 × 10^7^ LV-miR-665-ZR-75-30 cells or LV-miR-control-ZR-75-30 cells separately. Then the subcutaneous xenografts were observed and measured every 3 days. After 24 days, the mice were euthanized and the formed tumors were resected and weighted. Tumor size was measured using calipers, and tumor volumes were calculated according to the formula: *V* = a*b^2^/2, where *a* and *b* are the largest and shortest diameter in mm, respectively. Then, the resected tumors were fixed in 10% formalin and embedded in paraffin blocks for pathological examination.

Metastasis assays: 16 male BALB/c nude mice (3–4 week old) were randomized into two groups. Then, 100 μL of cell suspension containing 1 × 10^7^ LV-miR-665-ZR-75-30 cells or LV-miR-control-ZR-75-30 cells was injected intravenously through the tail vein into each mouse. The experiment was terminated after 8 weeks, the mice were euthanized and the lungs were removed and fixed with 10% formalin. Subsequently, consecutive tissue sections were made from each block of the lung. The sections were stained with hematoxylin-eosin staining (H&E).

The animals were housed under standard conditions and were supplied with food and water ad libitum according to the institutional guidelines for animal care. The experiments were performed in accordance with the guidelines of the Laboratory Animal Ethics Committee of Sun Yat-Sen University.

### IHC staining

IHC analysis and qualification of NR4A3 expression were performed using a standard streptavidin–biotin–peroxidase complex method^24^. Briefly, fresh tissue specimens were fixed in 10% formaldehyde and routinely processed for paraffin embedding. Then these blocks were cut into 4 μm thick sections. Hydrogen peroxide (3%) in methanol was used to block endogenous peroxidase activity. After antigen retrieval with microwave heating, the sections were incubated with rabbit polyclonal NR4A3 antibody (1:100, Signalway Antibody LLC, Maryland, USA) overnight at 4 °C. Then, the sections were incubated with HRP-conjugated anti-rabbit IgG secondary antibody (KeyGEN, Guangzhou, China) for 30 min at room temperature, followed by development using 3, 5-diaminobenzidine (DAB, KeyGEN, Guangzhou, China) substrate and counterstaining with hematoxylin for the nuclei.

### Gene set enrichment analysis

The information of BC samples with highest or lowest levels of miR-665 (*n* = 10, respectively) in TCGA database were downloaded and the fold change between the two groups was more than eight times. Gene set enrichment analysis was carried out using nonparametric scores with all default settings.

### Online bioinformatics analysis

In the Kaplan–Meier plotter database (http://kmplot.com/analysis/), we analyzed the relationship between gene expression (miR-665 and NR4A3) and BC patients’ survival including OS, relapse-free survival (RFS) and DMFS with Kaplan–Meier estimator. Putative miR-665 target genes were predicted using the miRNA target prediction algorithm TargetScan (http://www.targetscan.org/) and miRDB database (http://www.mirdb.org/).

### Statistical analysis

Chi squared (*χ*^2^) test was used for analyzing the relationships between miR-665 expression and clinicopathological features of patients with breast cancer. Survival curves were plotted using the Kaplan–Meier method and compared using a log-rank test. Student’s *t* test was used to test the differences of clinical features between two groups. All of statistic analyses and plots were performed in SPSS version 17.0 software (SPSS, Inc., Chicago, IL, USA) and GraphPad Prism 5 software (GraphPad Software, San Diego, CA, USA). ImageJ software was used to analyze cell migration, invasion and protein expression.

## Results

### miR-665 expression is significantly elevated in BC tissues and cell lines

**Fig. 1:**
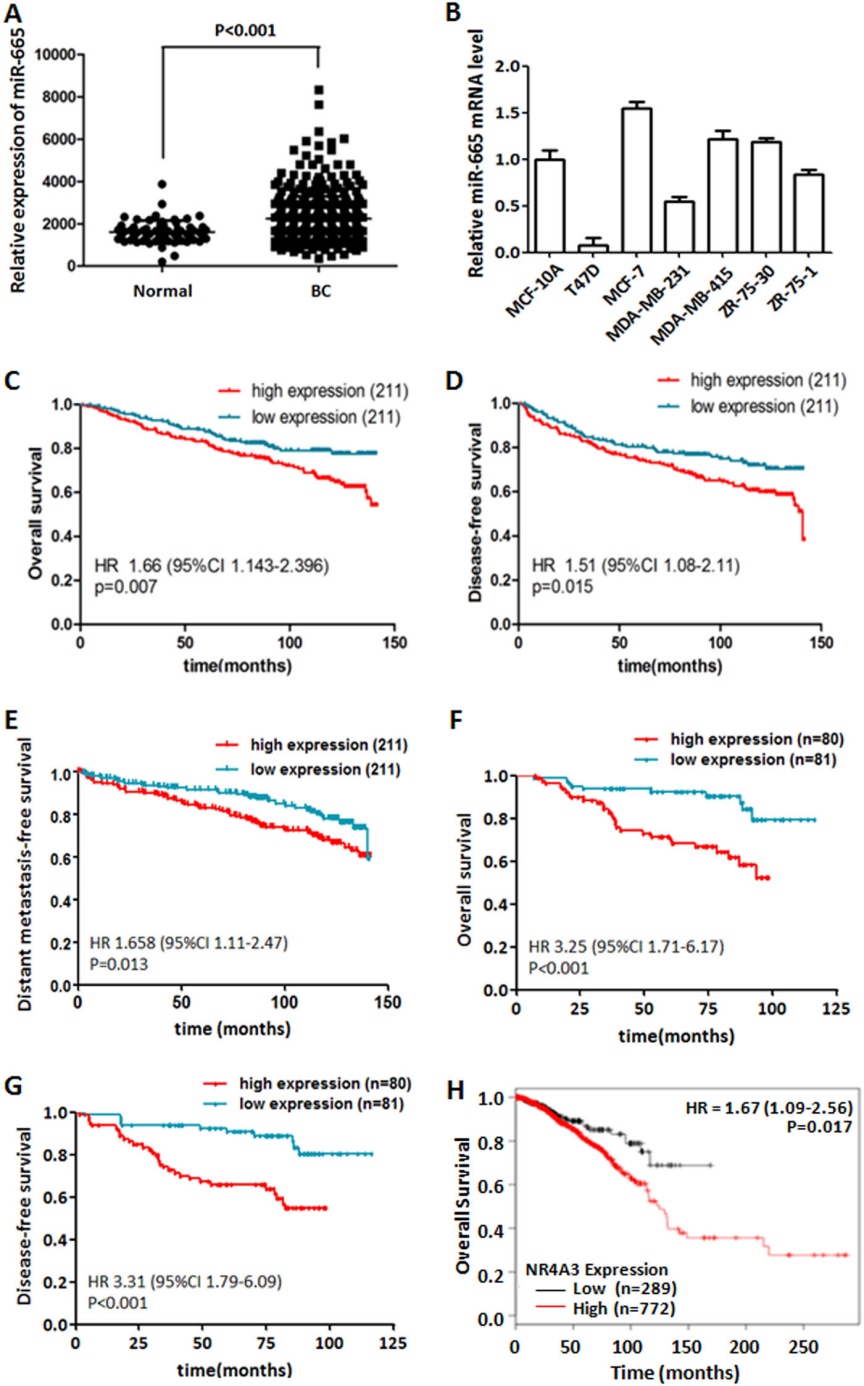
miR-665 is significantly overexpressed in breast cancer (BC) tissues and cell lines and associated with poor survivals of BC patients. **a** The relative mRNA expression of miR-665 detected by microarray in 422 BC tissues was significantly higher than that in 31 adjacent nontumor breast tissues (*P* < 0.001, independent Student’s *t* test). **b** The relative expression levels of miR-665 measured by qRT-PCR in MCF-7, MDA-MB-415, ZR-75-30, T47D, MDA-MB-231, ZR-75-1 BC cell lines, and an immortalized breast epithelial cell line, MCF-10A. Relative miR-665 levels were determined by normalizing to U6 messenger RNA (mRNA) levels. **c**–**e** Patients with high level of miR-665 had a remarkably worse OS (**c**), DFS (**d**), and DMFS (**e**) than those with low level of miR-665 in SYSUCC cohort (422 cases), which was analyzed by Kaplan–Meier curve (log-rank test). **f**, **g** Patients with miR-665 high-expression also had a notably poorer OS (**f**) and DFS (**g**) than those with miR-665 low-expression in FAHGMU cohort (161 patients). **h** That patients with high miR-665 expression had a poor overall survival was confirmed in 1061 BC patients obtained from Kaplan–Meier plotter database

In order to identify the miRNA expression profile in BC, total RNAs from 422 breast cancer specimens and 31 noncancerous breast tissues obtained from SYSUCC were detected using our custom miRNA microarray containing 1849 probes^20^. The result showed that 399 of the probed 1849 miRNAs were differentially expressed between BC and noncancerous tissues, in which 193 miRNAs were upregulated and 206 downregulated in BC tissues (detailed results will be published in another paper). Among the upregulated miRNAs, miR-665 was significantly upregulated in the BC tissues compared with non-tumor tissues examined by our microarray system (*P* < 0.001) (Fig. 1a). Next, we investigated the expression levels of miR-665 in several BC cell lines with real-time RT-PCR analysis, and found a markedly higher level of miR-665 in MCF-7, MDA-MB-415, and ZR-75-30 cell lines, and lower levels in T47D, MDA-MB-231, and ZR-75-1 cell lines when the data were normalized to the normal breast epithelial cell line, MCF-10A (Fig. 1b), indicating that miR-665 is overexpressed in 50% (3/6) of our examined BC cell lines.

### Overexpression of miR-665 is associated with poor survival and tumor metastasis in BC patients

In order to evaluate the clinical significance of the overexpressed miR-665, we first analyzed the relationship between clinical characteristics and miR-665 expression in SYSUCC cohort. Patients were divided into high- or low-level group by the median value of miR-665 expression in BC tissues. The result is presented in Table 2, which shows that high expression of miR-665 is significantly associated with higher T stage (*P* = 0.035), N stage (*P* = 0.041), TNM stage (*P* < 0.001), and postoperative metastasis (*P* = 0.007). Next, we examined the association of miR-665 expression with the survival of BC patients. Patients with high miR-665 expression in their tumors had much poorer OS, DFS, and DMFS than those with low miR-665 expression (Fig. 1c–e). Cox regression analysis of the microarray data indicated that miR-665 expression and TNM staging were independent prognostic indicators for OS and DFS of BC patients (Table 3). All these results suggest that miR-665 plays an important role in BC progression and metastasis.

**Table 2:** Association of miR-665 expression with clinicopathological characteristics in 422 BC patients

Characteristics	*n*	miR-665 expression level		*P* Value
		Low level	High level	
		*n* (%)	*n* (%)	
*Age*				
≥50	169	82 (38.9)	87 (41.2)	0.619
<50	253	129 (61.1)	124 (58.8)	
*Menopause*				
Yes	164	79 (37.4)	85 (40.3)	0.549
No	258	132 (62.6)	126 (59.7)	
*Pathological Grade*				
I	17	6 (2.8)	11 (5.2)	0.419
II	345	173 (82.0)	172 (81.5)	
III	60	32 (15.2)	28 (13.3)	
*Estrogen receptor*				
Positive	246	124 (58.8)	122 (57.8)	0.843
Negative	176	87 (41.2)	89 (42.2)	
*Progesterone receptor*				
Positive	234	118 (55.9)	116 (55.0)	0.845
Negative	188	93 (44.1)	95 (45.0)	
*Her-2*				
Positive	120	59 (28.0)	61 (28.9)	0.828
Negative	302	152 (72.0)	150 (71.1)	
*TNBC*				
Yes	46	27 (12.8)	19 (9.0)	0.211
No	376	184 (87.2)	192 (91.0)	
*T stage*				
T1–2	363	189 (52.1)	174 (47.9)	**0.035**
T3–4	59	22 (37.3)	37 (62.7)	
*N stage*				
N0–1	301	141 (46.8)	160 (53.2)	**0.041**
N2–3	121	70 (57.9)	51 (42.1)	
*Distant metastasis*				
No	372	195 (92.4)	177 (83.9)	**0.007**
Yes	50	16 (7.6)	34 (16.1)	
*TNM stage*				
I	71	32 (66.7)	16 (33.3)	**<0.001**
II	214	79 (45.7)	94 (54.3)	
III	137	63 (60.6)	41 (39.4)	

The bold values are less than 0.05, which have statistic significance

**Table 1:** Clinicopathological characteristics of patients with BC in the two cohorts

Characteristics	SYSUCC	FAHGMU
	*n* (%)	*n* (%)
*Age*		
≥50	169 (40.0)	84 (52.2)
<50	253 (60.0)	77 (47.8)
*Menopause*		
Yes	164 (38.9)	75 (46.6)
No	258 (61.1)	86 (53.4)
*Pathological Grade*		
I	17 (4.0)	7 (4.3)
II	345 (81.8)	134 (83.2)
III	60 (14.2)	20 (12.5)
*Estrogen receptor*		
Positive	246 (58.3)	103 (64.0)
Negative	176 (41.7)	58 (36.0)
*Progesterone receptor*		
Positive	234 (55.5)	101 (62.7)
Negative	188 (44.5)	60 (37.3)
*Her-2*		
Positive	120 (28.4)	104 (64.6)
Negative	302 (71.6)	57 (35.4)
*TNBC*		
Yes	46 (10.9)	13 (8.1)
No	376 (89.1)	148 (91.9)
*T stage*		
T1–2	363 (86.0)	138 (27.5)
T3–4	59 (14.0)	23 (28.0)
*N stage*		
N0–1	301 (71.3)	131 (81.4)
N2–3	121 (28.7)	30 (18.6)
*Distant metastasis*		
Yes	50 (11.8)	NA
No	372 (88.2)	NA
*TNM stage*		
I	71 (73.2)	26 (26.8)
II	214 (69.7)	93 (30.3)
III	137 (76.5)	42 (24.5)

To confirm the clinical significance of miR-665 overexpression, we detected miR-665 expression with qRT-PCR in FAHGMU cohort (161 BC samples) and analyzed its correlation with survival. Survival analysis indicated that high miR-665 expression is related with poor OS and DFS (Fig. 1f, g), and Cox regression analysis showed that miR-665 is an independent prognostic factor for OS and DFS (Table 4), which is consistent with the results obtained from SYSUCC cohort. These results demonstrate that miR-665 high-expression not only was significantly correlated with poor survival but also with elevated risk of metastasis.

**Table 4:** Cox regression analysis of characteristics associated with survival of BC patients in FAHGMU cohort

Clinical characteristics	Univariate Cox regression analysis		Multivariate Cox regression analysis	
	HR (95% CI)	*P* Value	HR (95% CI)	*P* Value
*Overall survival*				
miR-665 (high vs. low level)	3.489 (1.692–7.196)	**0.001**	3.311 (1.577–6.591)	**0.002**
Age (≥50 vs. <50)	1.433 (0.747–2.746)	0.279		
Menopause (yes vs. no)	1.413 (0.746–2.676)	0.289		
Pathological Grade (III vs. I and II)	1.314 (0.549–3.145)	0.539		
Her-2 (positive vs. negative)	0.774 (0.404–1.484)	0.440		
PR (positive vs. negative)	1.103 (0.564–2.156)	0.774		
ER (positive vs. negative)	0.771 (0.405–1.470)	0.430		
TNBC (positive vs. negative)	3.644 (1.594–8.331)	**0.002**	2.707 (1.139–6.433)	**0.024**
TNM stage (III vs. II vs. I)	2.131 (1.248–3.638)	**0.006**	1.112 (0.565–2.191)	0.758
*Disease-free survival*				
miR-665 (high vs. low level)	3.546 (1.779–7.068)	**<0.001**	3.278 (1.619–6.636)	**0.001**
Age (≥50 vs. <50)	1.383 (0.747–2.562)	0.302		
Menopause (yes vs. no)	1.249 (0.681–2.290)	0.473		
Pathological Grade (III vs. I & II)	1.685 (0.780–3.640)	0.184		
Her-2 (positive vs. negative)	0.764 (0.413–1.415)	0.392		
PR (positive vs. negative)	1.026 (0.546–1.929)	0.937		
ER (positive vs. negative)	0.814 (0.439–1.508)	0.513		
TNBC (positive vs. negative)	3.568 (1.650–7.715)	**0.001**	2.739 (1.224–6.131)	**0.014**
T stage (3–4 vs. 1–2)	3.256 (1.690–6.276)	**<0.001**	3.564 (1.590–7.988)	**0.002**
N stage (2–3 vs. 0–1)	2.332 (1.212–4.488)	**0.011**	2.430 (1.087–5.435)	**0.031**
TNM stage (III vs. II vs. I)	2.007 (1.211–3.326)	**0.007**	1.107 (0.553–1.870)	0.957

The bold values are less than 0.05, which have statistic significance

Next, we wondered if the overexpressed miR-665 had the same clinical significance in BC patients from different geographical areas of the world. To this purpose, we extracted the miR-665 data from Kapla–Meier plotter database (http://kmplot.com/analysis/) and assessed its relationship with the survival of BC patients. In this database, KM plot results obtained from 1061 BC patients in TCGA dataset and 1262 BC patients in METABRIC dataset indicated that patients with a high level of miR-665 had significantly poor OS than those with a low level (Fig. 1h and Fig. S1A), which suggested that overexpressed miR-665 is ubiquitously correlated with poor survival of BC patients from different parts of the world. Furthermore, we found that miR-665 was only associated with poor survival in BC patients with estrogen receptor (ER)-positive tumor but not in patients with ER-negative tumor in both TCGA and METABRIC databases (Fig. S1B–E). Altogether, these results indicate that miR-665 is an onco-miRNA that mainly promotes the progression and metastasis of ER-positive BC.

### miR-665 promotes BC cell proliferation and tumor growth

**Fig. 2:**
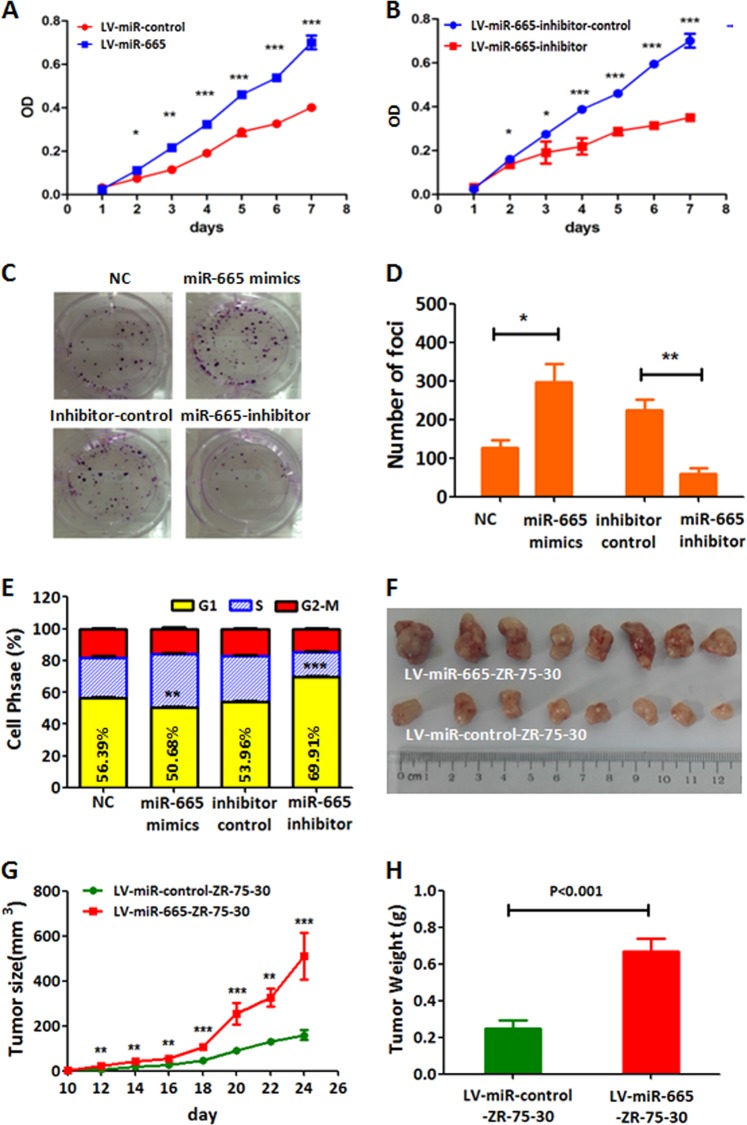
miR-665 promotes BC cells proliferation and tumor growth. **a** Compared with the control MCF-7 cells stably expressing a scramble oligonucleotide, the MCF-7 cells stably expressing miR-665 have a much higher proliferation rate in cell counting kit-8 (CCK-8) assay. **b** In CCK-8 assay, MCF-7 cells stably expressing miR-665 inhibitor showed a significantly lower cell proliferation rate compared with the control MCF-7 cells. **c** In plate colony formation assay, the MCF-7 cells transfected with miR-665 mimics exhibited much more colonies while the MCF-7 cells transfected with miR-665 inhibitor displayed much less colonies compared with their control cells, respectively. **d** The histogram is used to compare the colony numbers (average + standard deviation (SD)) from MCF-7 cells with different treatments in (**c**). **e** In the flow cytometry assay, the MCF-7 cells transfected with miR-665 mimics had the decreased G1 phase cells and the increased S phase cells while the MCF-7 cells transfected with miR-665 inhibitors were arrested in G1 phase (G1 phase cells were increased and S phase cells were decreased) compared with their control cells, respectively. **f** The subcutaneous xenograft tumors generated from LV-miR-665-ZR-75-30 cells (highly expressing miR-665) were significantly larger than those from control BC cells (LV-miR-control-ZR-75-30) in nude mice. **g** The tumor growth curves and **h** the average tumor weights and SDs in the two nude mice groups injected with ZR-75-30 cells stably expressing miR-665 or control oligonucleotide. (*P < 0.05, **P < 0.01, ***P < 0.001)

To substantiate the oncogenic role of miR-665 in BC, we constructed BC cells stably overexpressing or downregulating miR-665 by lentivirus transduction with miR-665 mimics or inhibitor in MCF-7, MDA-MB-231, and ZR-75-30 BC cell lines and analyzed cell proliferation by CCK-8 assay. Quantitative RT-PCR showed that miR-665 is overexpressed in cells stably transducted with miR-665 mimics and less-expressed in cells stably transducted with inhibitor when compared with their corresponding control cells (Fig. S2A–C). CCK-8 assay exhibited that the upregulation of miR-665 markedly promoted proliferation of MCF-7, MDA-MB-231, and ZR-75-30 cells, whereas the downregulation of miR-665 inhibited cell growth of these cell lines (Fig. 2a, b and Fig. S2D–G). Similarly, upregulation of miR-665 enhanced colony formation of BC cell, whereas downregulation of miR-665 reduced oncogenic colony formation in MCF-7 cells compared with the corresponding control cells (Fig. 2c, d). These results suggested that miR-665 could promote cell proliferation and drive oncogenic growth in BC cells. To investigate how miR-665 enhance BC cell proliferation or growth, we examined the effects of miR-665 on cell cycle and apoptosis by flow cytometry. The results revealed that the expressed miR-665 accelerated cell cycle progression by increasing the number of cells in S phase, whereas the downregulation of miR-665 markedly arrested cell cycle progression by raising the number of cells in G1 phase when compared with the corresponding control cells (Fig. 2e). In addition, flow cytometry analysis also revealed that ectopic expression of miR-665 inhibited cell apoptosis while the blockage of miR-665 expression increased cell apoptosis significantly (Fig. S2H, I). These in vitro results demonstrated that miR-665 promoted cell proliferation by accelerating G1/S phase transition and inhibiting apoptosis.

To further confirm the in vitro oncogenic role of miR-665, we subcutaneously injected miR-665 stably overexpressing cells (LV-miR-665-ZR-75-30 cells) and control cells into nude mice, respectively, to observe the effect of miR-665 on tumor growth in vivo. At 24 day after inoculation of tumor cells, the mice were euthanized and tumors were removed and measured. The results indicated that the volume and weight of tumors formed from LV-miR-665-ZR-75-30 cells (*n* = 8) were significantly higher than those formed from control cells (*n* = 8) (*P* < 0.05, Fig. 2f–h), which demonstrated that miR-665 could also promote tumor growth in vivo.

### miR-665 is required for cell migration, invasion, lung metastasis and EMT in BC

**Fig. 3:**
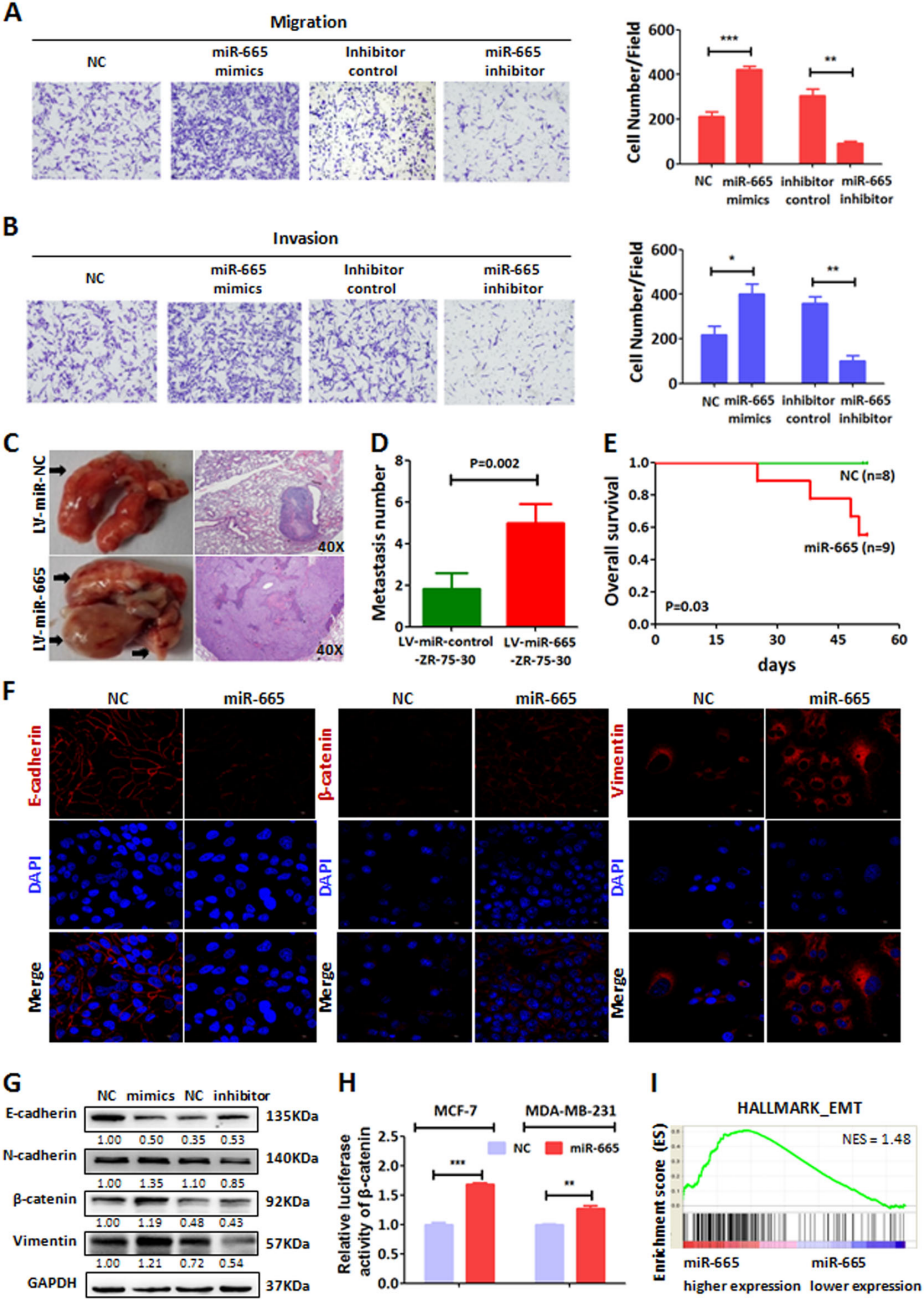
miR-665 promotes EMT, migration, invasion, and metastasis of BC cells in vitro and in vivo. **a** In transwell migration assay, MDA-MB-231 cells with transient transfection of miR-665 mimics showed more migrated cells and the cells with transient transfection of miR-665 inhibitors displayed much less migrated cells compared with their control cells, respectively (left panel); in the histogram, the numbers of migrated cells with different treatments in the left panel were compared (right panel). **b** In transwell invasion assay, MDA-MB-231 cells transfected with miR-665 mimics had more invaded cells and the cells transfected with miR-665 inhibitor had fewer invaded cells compared with their control cells, respectively (left panel); in the histogram, the numbers of invaded cells with different treatments in the left panel are compared (right panel). **c** Left column, the ZR-75-30 cells expressing miR-665 or control oligonucleotide were injected into the tail veins of nude mice, and the lungs were resected at 50 day, in which the lung sample from mice with cells expression miR-665 had more metastatic nodules than those with control cells; right column, lung sections stained with haematoxylin and eosin exhibited a small metastatic focus from LV-miR-control-ZR-75-30 cells and a large tumor from LV-miR-665-ZR-75-30 cells magnification (×40). **d** The histogram was used to compare the average numbers of metastatic nodules in the lungs of the two mouse groups. (**e**) Survival curves of the two nude mouse groups, the mice injected with LV-miR-665-ZR-75-30 cells had significantly worse survival than the mice injected with LV-miR-control-ZR-75-30 cells. **f** In Immunofluorescence assay, MCF-7 cells treated with miR-665 NC or miR-665 mimics for 48 h, and the EMT protein markers were detected with the corresponding antibodies which is shown in red and DAPI is shown in blue. **g** In western blotting analysis, overexpression of miR-665 (mimics) decreased the level of epithelial protein maker (E-cadherin) and increased the levels of mesenchymal protein markers (N-cadherin, Vimentin, and β-catenin) in MCF-7 cells and the reverse results were observed in the cells with miR-665 downregulation (miR-665 inhibitor). **h** A dual-reporter (Gaussia Luciferase (GLuc) and Secreted Alkaline Phosphatase) system containing β-catenin promoter was co-transfected with miR-665 NC or miR-665 mimics into MCF-7 or MDA-MB-231 cells. Luciferase activity was detected after incubation for 48 h and data represent mean ± SD compared to control cells (*n* = 3), which indicated that when miR-665 was ectopically expressed in BC cells, β-catenin transcriptional activity was increased. **i** Gene set enrichment analysis (GSEA) based on ten patients with low-miR-665 expression (blue) and ten those with high-miR-665 expression (red) showed that miR-665 high-expression was involved in the in EMT pathway activation. NES means normalized enrichment score. ^*^P < 0.05, ^**^P < 0.01, ^***^P < 0.001, independent Student’s *t* test

The result of the clinical analysis above shows that miR-665 expression is associated with distant metastasis of BC. Therefore, we want to examine whether miR-665 can advance BC metastasis in vitro and in vivo. To this end, we first examined the effect of miR-665 on migration and invasion of BC cells with transwell assay. The number of migrating or invading MDA-MB-231 cells stably expressing miR-665 was much larger than that of the control MDA-MB-231 cells, whereas the number of migrated or invaded MDA-MB-231 cells stably expressing miR-665 inhibitor was much less than that of control cells (Fig. 3a, b), which suggested that miR-665 could enhance the metastatic potential of BC in vitro.

Next, we want to test if miR-665 increases the metastatic capability of BC cells in vivo. The LV-miR-665-ZR-75-30 cells stably expressing miR-665 and the control cells were separately injected into nude mice through the tail vein. At 52 day after injection of BC cells, the remaining alive mice were euthanized. After removal, the lungs were fixed with 10% formalin and embedded in paraffin. Under a microscope, the metastatic lesions in the lung sections were calculated. The result showed that lung metastatic lesions in mice (*n* = 8) injected with miR-665-expressing cells were significantly more than those in mice (*n* = 9) with control cells (*P* < 0.01, Fig. 3g, h). Furthermore, 4 mice injected with miR-665-expressing cells died as a consequence of metastatic lesions in the lung within 52 days whereas none of the mice with control cells died in the same period (Fig. 3i), indicating that miR-665 expression enhances metastasis of BC cells and results in poor survival of mice. Altogether, the above results demonstrate that miR-665 is required for BC cell migration and invasion in vitro, and metastasis in vivo, which is consistent with the clinical results. In general, epithelial–mesenchymal transition (EMT) is known as the first step of “invasion-metastasis cascade”^25^. Therefore, we asked if miR-665 could regulate EMT of BC cells. To this end, we first detected EMT protein markers by western blot analysis. The result showed that overexpressed miR-665 indeed increased the expression of mesenchymal markers including N-cadherin, β-catenin and Vimentin, and decreased epithelial marker E-cadherin in MCF-7 cells (Fig. 3g). To directly visualize the EMT, we performed immunofluorescence assay on EMT markers in BC cells. In MCF-7 cells with overexpressing miR-665, the levels of β-catenin and Vimentin were elevated and E-cadherin expression level was downregulated (Fig. 3f), suggesting that miR-665 may induce EMT before promoting the migration, invasion, and metastasis of BC cells. To further confirm that miR-665 expression induces EMT phenotype, we performed a dual-luciferase reporter assay on the promoter of β-catenin to verify miR-665 promoting β-catenin transcription. The result showed that the luciferase activity of the cells with miR-665 overexpression was significantly higher than that in the control cells in both MCF-7 and MDA-MB-231 cells (Fig. 3h), suggesting that overexpressed miR-665 promotes transcriptional activity of β-catenin in BC cells. To theoretically verify that miR-665 can activate EMT pathway, we carried out gene set enrichment analysis (GSEA) based on ten pairs of BC samples with high and low miR-665 expressions (approximately eight-fold difference in average expression levels) obtained from TCGA database. Based on hallmark gene set, GSEA showed enrichment of genes involving in EMT in BC samples (Fig. 3i), which is consistent with the above experiment results.

### NR4A3 is the target of miR-665 in breast cancer

Given the above significant effects induced by miR-665 in BC, we first need to decipher the target of miR-665 in BC. Thus, we set out to search the possible targets of miR-665 on the web-based resources (https://mirmap.ezlab.org/app/ and http://www.mirdb.org). In both of databases, there are 459 shared predicted target genes of miR-665. According to miR-665 promotion of BC growth and metastasis, we selected 17 possible target genes to test which will be regulated by miR-665. In the BC cells overexpressing or underexpressing miR-665, we examined the mRNA expression of the 17 genes with qRT-PCR. The result shows that of the 17 genes, only mRNA levels of NR4A3 and transforming growth factor beta receptor 2 (TGFBR2) were both decreased in cells overexpressing miR-665 and both increased in cells underexpressing miR-665 when compared with their corresponding control cells (Fig. S3B, C), which suggests that NR4A3 and TGFBR2 are the target of miR-665. Since TGFBR2 has been proved to be a target of miR-665 in pancreatic cancer and other cancer cells^18,26^, we only focued on the novel target, NR4A3, of miR-665 in BC.

**Fig. 4:**
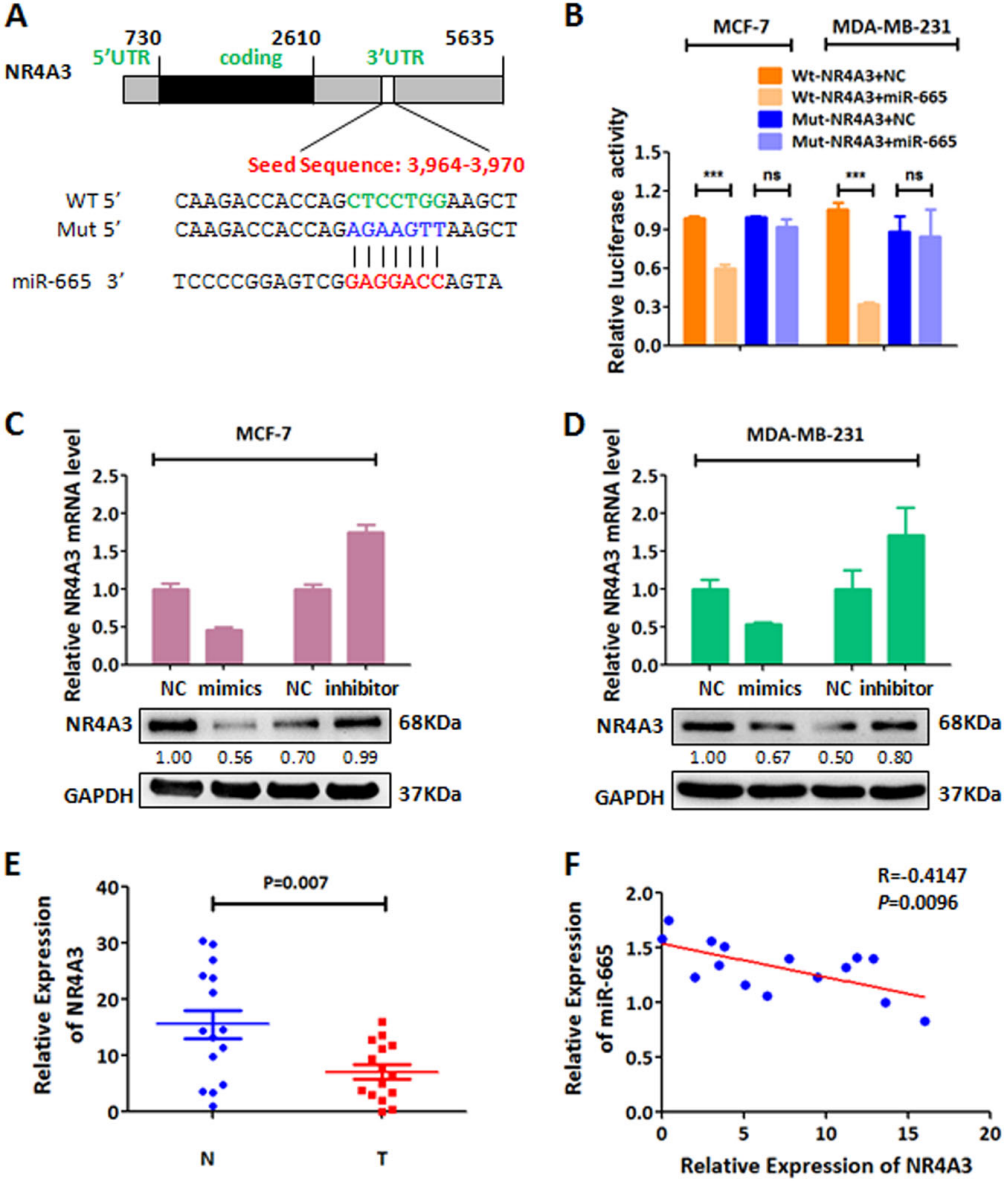
miR-665 targets NR4A3 in BC cells. **a** The binding seed sequence (in green) of miR-665 in 3′UTR of NR4A3 mRNA as predicted by the TargetScan algorithm, and the artificially mutated seed sequence (in blue) in the same 3′UTR, and the seed sequence in mir-665 (in red). **b** In dual-luciferase assay, MCF-7 or MDA-MB-231 cells co-transfected with reporter vector containing wild type 3′UTR of NR4A3 and miR-665 mimics showed a significant reduction in luciferase activities compared with the control cells co-transfected with the wild-type 3′UTR and control oligonucleotide (NC), while in these cells co-transfected with the mutated 3′UTR of NR4A3 and miR-665 mimics, there was no reduction in luciferase activities compared with the control cells. **c**–**d** In qRT-PCR and western blotting analysis, MCF-7 (**c**) and MDA-MB-231 (**d**) cells transfected with miR-665 mimics exhibited low mRNA (upper panel) and protein (lower panel) levels of NR4A3; when these cells were transfected with miR-665 inhibitor, the mRNA (upper panel) and protein (lower panel) levels of NR4A3 were increased compared with the control cells. **e** The relative mRNA expression of NR4A3 detected by qRT-PCR was significantly lower in BC tissues (15 samples) in comparison with normal breast tissues (15 samples, *P* < 0.01, independent Student’s *t* test). **f** An inverse correlation was found between miR-665 expression and NR4A3 mRNA expression in 15 BC tissues, as examined by qRT-PCR (*R* = −0.4147, *P* = 0.001)

Bioinformatics analysis showed that there is one seed sequence between 3′ UTR of NR4A3 and miR-665 (Fig. 4a). To test if miR-665 can bind to 3′ UTR of NR4A3 via the seed sequences, we constructed wild type and mutated seed sequences in the 3′ UTR of NR4A3 mRNA (Fig. 4a). The 3′ UTR sequences was cloned into the Firefly luciferase reporter plasmid. Luciferase reporter experiment displayed that when MCF-7 or MDA-MB-231 cells were co-transfected with miR-665 mimics and the wild-type 3′ UTR, the cells had significantly lower relative luciferase activity than the cells with miRNA scramble controls and the wild-type 3′ UTR; when BC cells were co-transfected with miR-665 mimics and the mutated 3′ UTR, the cells had nearly the same relative luciferase activity as the control cells (Fig. 4b), which demonstrate that wild type miR-665 can bind to the seed sequences of the 3′ UTR of NR4A3 mRNA to inhibit translation and activity of luciferase whereas it can not bind to the mutated seed sequences so that luciferase activity is not affected. Furthermore, we employed qRT-PCR and western blot to examine whether the mRNA and protein of NR4A3 were inhibited by miR-665. The result reveals that the cells overexpressing miR-665 show an obvious downregulation of NR4A3 mRNA and protein whereas the cells underexpressing miR-665 exhibit upregulated levels of NR4A3 mRNA and protein when compared with the corresponding control cells in MCF-7 and MDA-MB-231 cells (Fig. 4c, d). Altogether, these results demonstrate that NR4A3 is a direct target of miR-665.

To further confirm that NR4A3 is a target of miR-665 in BC tissues, we explored the relationship between the expression levels of miR-665 and its target gene in BC samples. First, we used qRT-PCR to examine the RNA expression levels of miR-665 and NR4A3 in tumor tissues of BC patients. The result showed that NR4A3 mRNA levels in clinical BC tissues (*N* = 15) is significantly lower than the normal breast tissues (*N* = 15) (Fig. 4e) and that the mRNA level of NR4A3 was reversely correlated with the expression levels of miR-665 in these samples (*R* = −0.4147, *P* = 0.001) (Fig. 4f). Second, we investigated NR4A3 expression with immunohistochemical staining in xenograft tumors generated from BC cells overexpressing miR-665 (LV-miR-665-ZR-70-30 cells), and found that NR4A3 was downregulated in these tumors compared with that in the control tumors generated from LV-miR-control-ZR-70-30 cells (Fig S4A). These results clearly suggest that NR4A3 is the target of miR-665 in BC.

### miR-665 promotes BC invasion and metastasis by inhibiting NR4A3

**Fig. 5:**
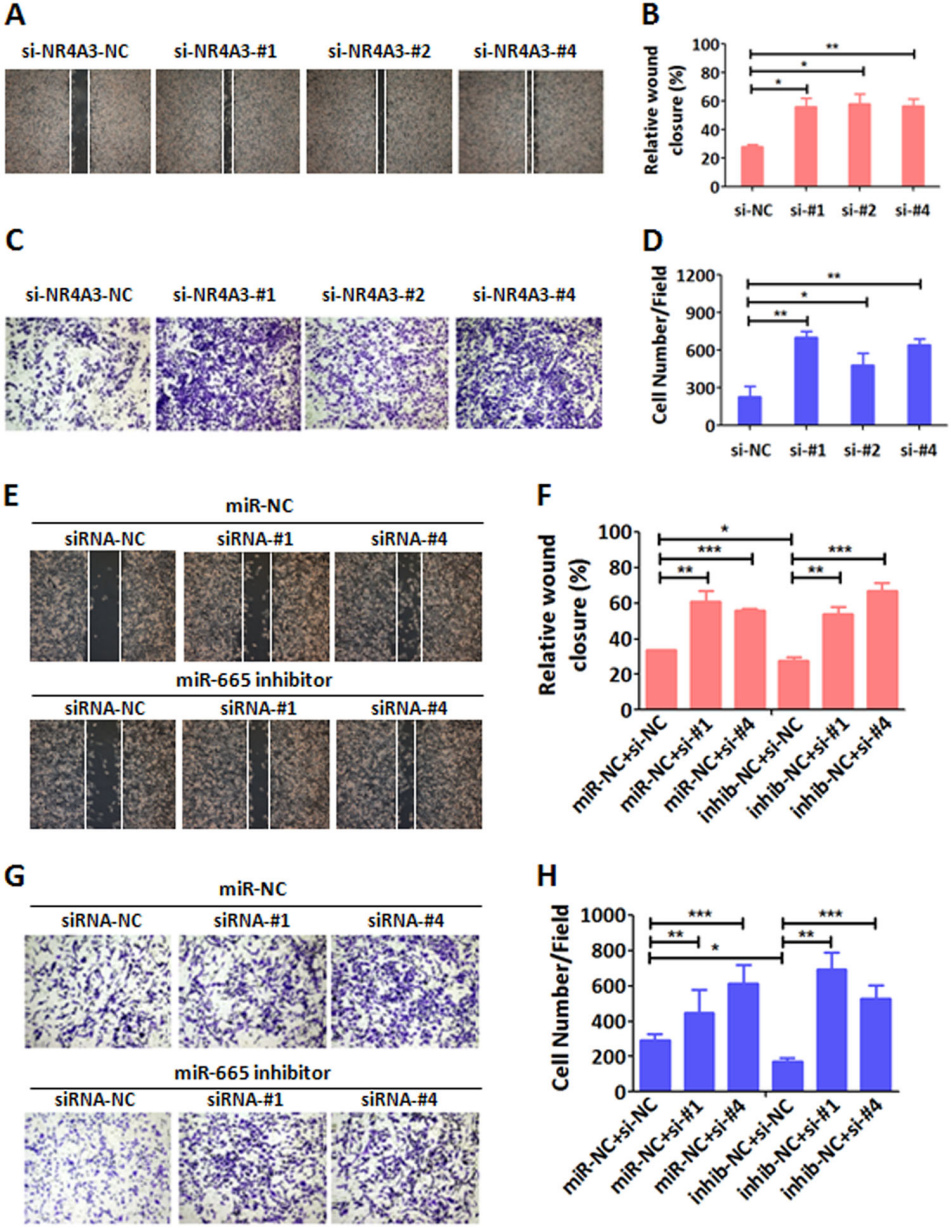
miR-665 enhances migration and invasion of BC cell by targeting NR4A3. **a** In scratch wound healing assay, MDA-MB-231 cells with transient transfection of NR4A3 siRNA #1, #2, #4 showed a significantly accelerated wound healing compared with the control cells treated by NR4A3 siRNA-NC. **b** In the histogram, the percentages of wound healing of the cells with different treatments in (**a**) were compared. **c** In transwell invasion assay, MDA-MB-231 cells with siRNA #1, #2, #4 against NR4A3 had more invaded cells. **d** In the histogram, the numbers of invaded cells with different treatments in (**c**) were compared. **e** In scratch wound healing assay, after MDA-MB-231 cells were treated with miR-665 inhibitor or miR-NC for 36 h and then with NR4A3 siRNA-NC, #1 or #4 for another 24 h, respectively, the cells with miR-665 inhibitor and siRNA #1 or #4 had an accelerated (rescued) wound healing compared with those with miR-665 inhibitor and siRNA-NC. **f** In the histogram, the percentages of the wound healing in the cells with different treatments in (**e**) were compared. **g** In transwell invasion assay, after MDA-MB-231 cells were transfected with miR-665 inhibitor or miR-NC for 36 h and then with NR4A3 siRNA-NC, #1 or #4 for another 24 h, respectively, the cells with miR-665 inhibitor and siRNA #1 or #4 had a greater number of invaded cells (rescued) compared with those with miR-665 inhibitor and siRNA-NC. **h** In the histogram, the numbers of invaded cells with different treatments in (**g**) were compared. In this figure, results were expressed as the mean ± SD of three independent experiments. ^*^P < 0.05, ^**^P < 0.01, ^***^P < 0.001 by Student’s *t* test

**Fig. 6:**
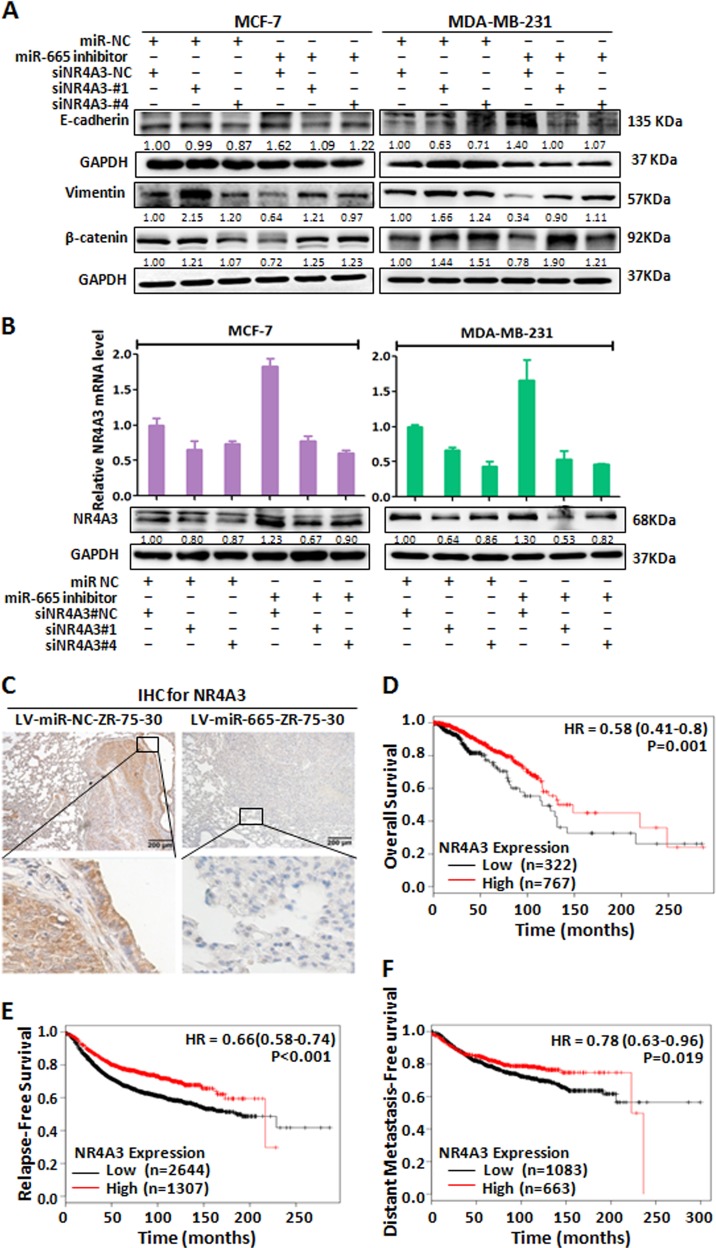
miR-665 promotes metastasis in BC by inhibition of NR4A3. **a** In MCF-7 and MDA-MB-231 cells, the treatment with miR-NC and siRNAs against NR4A3 decreased E-cadherin protein expression and increased the expression of Vimentin and β-catenin proteins compared with that with miR-NC and siRNA-NC; when the treatment with miR-665 inhibitor and siRNA-NC was employed, the expression pattern of E-cadherin, Vimentin and β-catenin proteins was inversed compared with that with miR-NC and siRNA-NC and that with miR-NC and siRNAs against NR4A3; when the treatment with miR-665 inhibitor and siRNAs against NR4A3 were sequentially employed, the expression pattern of the three proteins was restored (rescued) again compared with that with miR-NC and siRNA-NC and that with miR-NC and siRNAs against NR4A3, as detected by western blot. **b** The mRNA (upper panel) and protein (lower panel) levels of NR4A3 were decreased when MCF-7 and MDA-MB-231 cells treated with miR-NC and siRNAs against NR4A3 compared with that with miR-NC and siRNA-NC; when these cells were transfected with miR-665 inhibitor and siRNA-NC, NR4A3 expression was increased; when the cells were sequentially depleted of miR-665 by inhibitor and NR4A3 by siRNA, NR4A3 expression was downregulated again, as determined by qRT-PCR and western blot. **c** In IHC analysis, expression of NR4A3 was markedly downregulated in lung metastatic tumors in nude mice with injection of LV-miR-665-ZR-75-30 cells from tail veins compared with control metastatic tumors in mice with injection of LV-miR-NC-ZR-75-30 cells. **d**–**f** The BC patients with high NR4A3 expression had better overall survival (**d**), release-free survival (**e**), and distant metastasis-free survival (**f**), which were obtained from online Kaplan–Meier plotter database

It is known that NR4A3 is a transcriptional activator and plays important roles in many physiological and pathological processes^27^. More important, NR4A3 is involved in the development and progression of various tumors including the primary extraskeletal myxoid chondrosarcoma, lymphoma, acute myeloid leukemia and gastric cancer, and functions as a tumor suppressor^27–31^. Based on our above results that miR-665 targets NR4A3 and can promote EMT, migration, invasion, and metastasis of BC cells in vitro and in vivo, we argued that miR-665 advances the metastasis of BC by targeting NR4A3. To this end, using wound healing assay in MDA-MB-231, we first examined if silenced NR4A3 would promote BC migration as overexpressed miR-665 did. The result proved that the wound healing was significantly accelerated in NR4A3-silenced MDA-MB-231 cells when compared with the control cells (Fig. 5a, b). Secondly, matrigel invasion assay also showed that the number of invaded MDA-MB-231 cells with silenced NR4A3 was significantly more than that of invaded control cells (Fig. 5c, d). These results indicate that silenced NR4A3 induces the same phenotypes of BC cells as miR-665 expression does, suggesting that miR-665 promotes migration and invasion of BC cells by targeting NR4A3. To substantiate our hypothesis, we conducted a rescue experiment in MDA-MB-231 cells. The BC cells depleted of endogenous miR-665 by miR-665 inhibitor were incubated for 36 h followed by depletion of NR4A3 using a siRNA aginst NR4A3 for another 24 h. As shown in the above experiments, we demonstrated that depletion of endogenous miR-665 with miR-665 inhibitor significantly inhibited the migration and invasion of MDA-MB-231 cells compared with the control cells in wound healing assay and matrigel invasion assay (Fig. 5e–g), respectively. In this rescue experiment, when MDA-MB-231 cells were transfected with siRNA against NR4A3, the cell migration and invasion were increased compared with the control cells; when the cells were sequentially depleted of miR-665 and NR4A3, the inhibited cells again restored their motility and invasiveness by exhibiting accelerated wound healing and increased invasion compared with the corresponding control cells (miR-inhibitor + siRNA-NC treatment) (Fig. 5f, h). Furthermore, we conducted the same rescue experiment in MCF-7 and MDA-MB-231 cells and observed EMT changes with western blot. As showed in Fig. 6a, when miR-665 expression was inhibited with miR-665 inhibitor, the expression of mesenchymal markers (Vimentin and β-catenin) was decreased and epithelial marker (E-cadherin) was increased compared with the control treatment in MCF-7 and MDA-MB-231 cells; when NR4A3 was depleted with siRNAs, the expression pattern of the three EMT markers was inversed in these cells; when the expressions of miR-665 and NR4A3 were inhibited sequentially with miR-665 inhibitor and siRNA, the expression pattern of three EMT markers was rescued again, suggesting that miR-665 promotes EMT by targeting NR4A3. At the same time, we examined mRNA and protein expression of NR4A3 by qRT-PCR and western blot when the BC cells were treated with miR-665 inhibitor and siRNAs against NR4A3. The result shows that the mRNA and protein expression of NR4A3 were increased when BC cells were treated with miR-665 inhibitor, whereas they were decreased when these BC cells were transfected again with siRNAs against NR4A3 (Fig. 6b).

As mentioned above, overexpressed miR-665 promoted lung metastasis of BC cells in nude mice. We wondered if NR4A3 was involved in the metastasis of BC. Thus, we investigated NR4A3 protein level in the metastatic nodules with immunohistochemistry (IHC) and found that NR4A3 was significantly reduced in the lung metastatic BC nodules from BC cells with upregulated miR-665 (Fig. 6c). In the TCGA database, NR4A3 expression in breast cancers is remarkably reduced compared with that in normal breast tissues (Fig. S4B). Furthermore, we explored the KM Plotter Database and found that patients with high-expression of NR4A3 had significantly better OS, RFS, and DMFS than those with low expression of NR4A3 (Fig. 6d–f), suggesting that NR4A3 expression inhibits metastasis and progression of BC in these patients. Altogether, these results undoubtedly demonstrate that miR-665 promotes invasion and metastasis by inhibiting NR4A3.

### miR-665 enhances metastatic phenotype in BC via NR4A3/MEK pathway

**Fig. 7:**
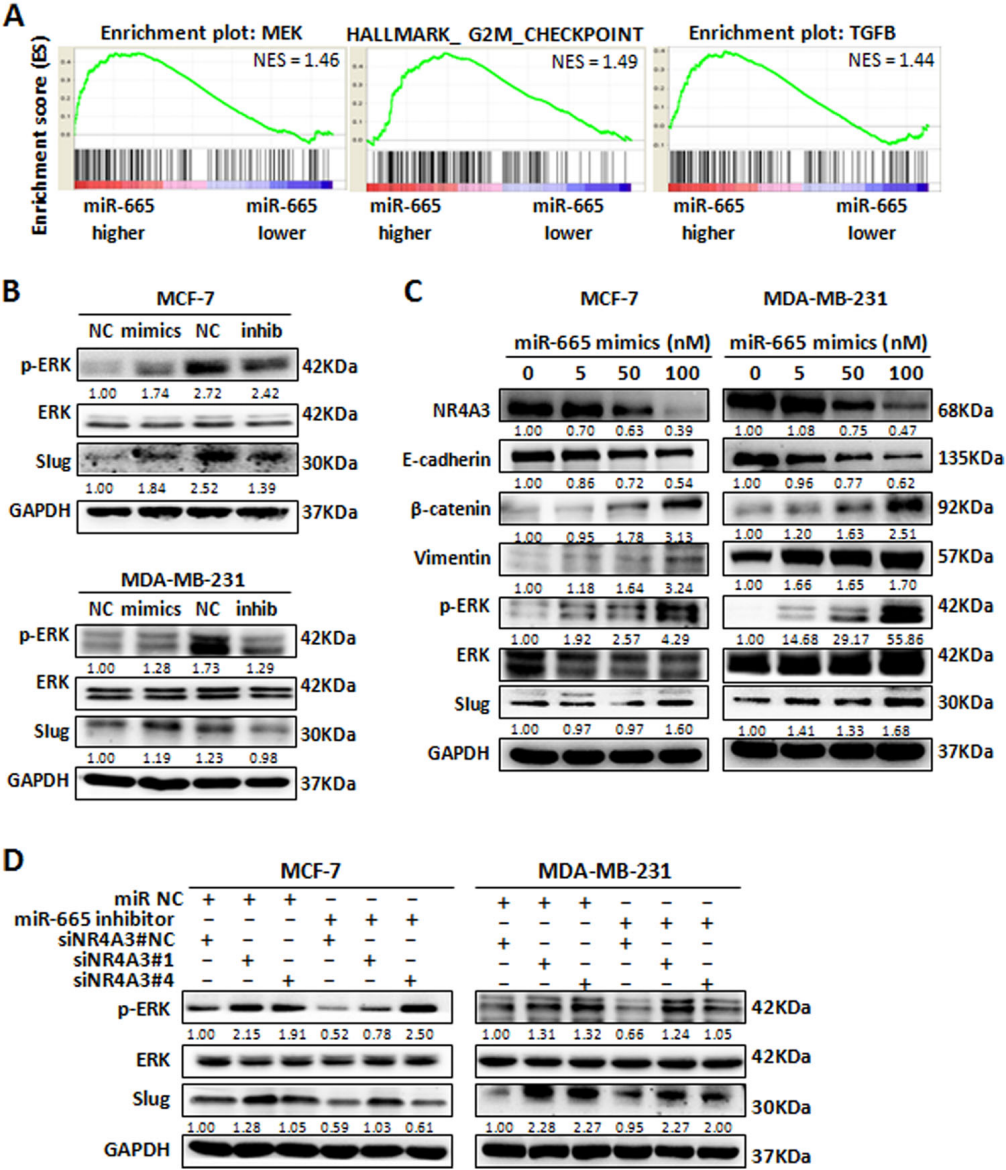
miR-665 expression contributes a more aggressive metastatic phenotype in BC via activation of NR4A3/MEK pathway. **a** GSEA analysis was performed in 10 pairs of BC tissues with miR-665 high expression (red) and low expression (blue) obtained from TCGA database, and the result showed that miR-665 high-expression was involved in activation of multiple signaling pathways, such as MEK, EMT, G2M, and TGFB pathways. NES normalized enrichment score. **b** In MCF-7 and MDA-MB-231 cells, western blotting analysis indicated that transfection of miR-665 mimics increased the level of ERK phosphorylation and Slug expression, whereas transfection of miR-665 inhibitors showed the reverse results compared with the corresponding control treatments. **c** In MCF-7 and MDA-MB-231 cells, when the cells were transfected with different doses of miR-665 mimics, the expression levels of NR4A3 and E-cadherin were gradually decreased while the expression of β-catenin, Vimentin, phosphorylated ERK (p-ERK), and Slug were increased following the increased doses of miR-665 mimics, as measured by western blot. **d** In MCF-7 and MDA-MB-231 cells, western blotting analysis showed that the protein levels of p-ERK and Slug were increased when the cells were depleted of NR4A3; when only miR-665 was downregulated by miR-665 inhibitor, the protein levels were decreased; when the cells were treated by sequential depletion of miR-665 and NR4A3, the p-ERK and Slug were increased (rescued) again. The values above the blot bands are the relative digital intesnsity of the bands

To understand the mechanism underlying miR-665 promotion of BC metastasis, we carried out GSEA as described above. Based on KEGG pathway and oncogenic signature/hallmark gene set, GSEA showed enrichment of genes involving in cell cycle, TGFB, MEK, and other pathways in high vs. low level of miR-665 in BC samples (Fig. 7a), the EMT and cell cycle of which have been verified in our above experiments. Since TGFB signaling and its upstream gene TGFBR2, which has been reported to be a target gene of miR-665 in pancreatic cancer and other cancer cells^18,26^, have been reported in BC^32,33^, we interested in whether miR-665 promotes BC metastasis by activating MEK signaling, which is reported to advance BC metastasis^34,35^. Thus, we investigated the downstream target ERK and Slug of MEK pathway in BC cells with miR-665 overexpression or underexpression. The result indicates that both Slug expression and ERK phosphorylation were increased in MCF-7 and MDA-MB-231 cells with miR-665 overexpression and decreased in BC cells with less expression of miR-665 when compared with the corresponding control cells (Fig. 7b), suggesting that miR-665 promotes migration, invasion and metastasis via activating MEK pathway. Then the key question is whether miR-665 activates MEK signaling by inhibiting NR4A3. To this purpose, we observed expression changes of NR4A3, Slug, and ERK under different doses of miR-665 mimics in BC cells. As expected, the expression of Slug, β-catenin, and Vimentin as well as ERK phosphorylation were gradually upregulated, and NR4A3 and E-cadherin were downregulated as the dose of miR-665 mimics was increased from 5, 50 to 100 nM (Fig. 7c). To further corroborate that miR-665 activates EMT and MEK pathways via inhibiting NR4A3, we conducted another rescue experiment: when only miR-665 was downregulated by miR-665 inhibitor, Slug expression and ERK phosphorylation were decreased; when only NR4A3 was downregulated by siRNAs, Slug expression and ERK phosphorylation also were inversed; when miR-665 and NR4A3 were downregulated sequentially, Slug expression and ERK phosphorylation were rescued again (Fig. 7d). The results indicate that NR4A3 represses MEK pathway including ERK phosphorylation and Slug expression, consistent with the report by Rodríguez group that NR4A3 inhibits ERK phosphorylation^36^. Altogether, our results demonstrate that upregulated miR-665 activates MEK pathway by inhibiting NR4A3 to promote EMT, migration, invasion, and metastasis of BC cells.

## Discussion

In this study, we profiled miRNA expression in 422 BC patients from Southern China using a custom microarray, and found that 399 miRNAs were differentially expressed between BC and noncancerous tissues. Among the upregulated miRNAs, miR-665 was highly expressed in BC tissues and associated with poor OS, DFS, and DMFS of BC patients. High-expression level of miR-665 also was found to be positively related to T stage, N stage, distant metastasis as well as TNM stage. The correlation between overexpressed miR-665 measured by qRT-PCR and poor survival was validated in 161 BC samples obtained from another medical center in South China. Furthermore, this correlation was corroborated in total 2323 BC patients from other parts of the world. The clinical data indicate that miR-665 is a pro-oncogenic regulator and promotes the development and progression of BC, especially tumor metastasis, suggesting that miR-665 is a potential biomarker for predicting survival and metastasis of BC.

Like many other miRNAs, miR-665 has been reported to show pro-oncogenic and tumor suppressive roles in different types of cancers because it suppress different target genes, oncogenes or tumor suppressors in different cancers^37^. As a tumor suppressor, miR-665 is downregulated in osteosarcoma tissues and represses metastasis of osteosarcoma cells by inhibiting Rab23 and TGFBR2^17,26^; miR-665 expression also is reduced in lung cancer and gastrointestinal stromal tumor cells and targets MAPK signaling pathways and CD34^38,39^; in pancreatic cancer cells, miR-665 is downregulated and inhibits oncogenic phenotype by targeting TGFBR1 and TGFBR2^18^; in ovarian cancer cells, miR-665 is downregulated and can suppress tumor growth and migration^40^; in mouse model, miR-665 can repress neuroblastoma cell growth by targeting c-myc and HDAC8^41^. However, miR-665 is also reported to be upregulated in HCC tissues, HCC cell lines and serum exosome of HCC patients, and the overexpressed miR-665 promotes HCC cell proliferation, migration, and invasion by inhibiting PTPRB^14,42^; a high level of miR-665 is associated with resistance to neoadjuvant radiochemotherapy in squamous cell carcinoma of the esophagus^43^; in nonsmall cell lung cancer, miR-665 is overexpressed in plasma extracellular vesicles of patients and lung cancer cell lines^15^, suggesting that miR-665 acts as an oncogene. In a word, miR-665 can act as either a tumor suppressor or an oncogene depending on the cancer type or the cellular content. Interestingly, one group reported that miR-665 was downregulated in 48 BC tissue samples compared with the corresponding adjacent breast tissues^19^, which is inconsistent with our result. The major reason might be the different sample sizes (422 BCs vs. 48 BCs) in our study and the others’.

In the present study, we demonstrated the oncogenic role of miR-665 in breast tumorigenesis and tumor metastasis by gain- and loss-of function experiment in vitro and in vivo. Notably, overexpression of miR-665 promotes migration and invasion of MDA-MB-231 cells and advances lung metastasis of BC cells in nude mice. GSEA analysis shows that high-expression of miR-665 enriched genes involving in EMT, suggesting that miR-665 can induce EMT. Therefore, we examined the transcriptional activity of β-catenin, a representational EMT marker, in BC cells expressing miR-665. The result shows that overexpressed miR-665 can upregulated the luciferase activity of β-catenin promoter in BC cells significantly, which suggests that by promoting β-catenin transcriptional activity, miR-665 may promote the EMT in BC. More important, we demonstrated that miR-665 promotes EMT, migration, invasion and metastasis of BC cells through targeting NR4A3. Firstly, ectopic expression of miR-665 decreases the mRNA and protein levels of NR4A3 in BC cells, and the downregulated miR-665 increases the expression of this gene. Secondly, miR-665 can bind to the 3′ UTR of NR4A3 as evidenced by luciferase reporter assay and bioinformatic analysis.

NR4A3 is a member of the steroid–thyroid hormone-retinoid receptor superfamily and is demonstrated to be a transcriptional activator that can efficiently bind to the NGFI-B response element (NBRE)^44,45^. Previous studies have suggested that NR4A3 acts as a tumor suppressor in tumorigenesis such as hematologic neoplasms, acute myeloid leukemia, lymphoma, and gastric cancer by reducing cell viability and inducing cell apoptosis^27,30,31^. In breast cancer, NR4A3 is downregulated, and functions as a tumor suppressor^46^, which is consistent with our result. In TCGA database, NR4A3 expression also dramatically reduced in BC tissues compared with normal breast tissues (Fig. S5B). In the present study, we found that NR4A3 was downregulated whereas miR-665 was upregulated in BC tissues, which were significantly inversely correlated with each other. In in vitro experiment, downregulation of NR4A3 markedly restored the EMT, migration, and invasion of BC cells inhibited by miR-665 inhibitor; in BC metastasis model in nude mice, NR4A3 is significantly reduced in the metastatic nodules formed from BC cells with miR-665 overexpression (Fig. 6c), indicating that miR-665 promotes EMT, migration, invasion, and metastasis of BC cells by targeting NR4A3. To explore the underlying the mechanism, we conducted a GSEA in TCGA database, which showed that high expression of miR-665 enriched genes involving in MEK, and other pathways, of which MEK is reported to advance BC metastasis^34,35^. Therefore, we mainly investigated whether miR-665 could activate MEK signaling in BC cells. The result indicates that miR-665 can regulated both Slug expression and ERK phosphorylation of MEK signaling cascade in MCF-7 and MDA-MB-231 cells (Fig. 7b). Next, we demonstrated that NR4A3 expression inhibited MEK pathway including ERK phosphorylation and Slug expression in BC cells. Finally, we showed that miR-665 activates MEK pathway by inhibiting NR4A3. These results indicate that miR-665 activated MEK signaling pathway via inhibiting NR4A3, by which miR-665 promotes metastasis phenotype of BC cells.

In this study, we demonstrate for the first time that miR-665 expression is associated with metastasis and poor survival in BC patients and plays an oncogenic role in BC progression with clinical analysis and both in vivo and in vitro experiments. Furthermore, we elucidate that miR-665 promotes BC invasion and metastasis via targeting NR4A3 to activate MEK signaling pathway. Altogether, our study provides the evidence that miR-665 is a potential new biomarker for recurrence, metastasis, and survival, and therapeutic target in patients with BC.

## Supplementary information

Supplemental Materials
