# Supplementary material for: miR-665 expression predicts poor survival and promotes tumor metastasis by targeting NR4A3 in breast cancer
Source: Cell Death Dis. 2019 Jun 17;10(7):479. doi: 10.1038/s41419-019-1705-z (PMC6579763; doi:10.1038/s41419-019-1705-z)
Supplement: Supplementary file 1 — Supplemental Materials [file 41419_2019_1705_MOESM1_ESM.docx]

**Supplemental Materials**

**miR-665 promotes** **tumor metastasis by targeting NR4A3 and predicts poor survival in breast cancer patients**

**Xin-Ge Zhao^1,2#^, Jing-Ye Hu^3#^, Jun Tang^4^, Wei Yi^5^, Mei-Yin Zhang^1,2^, Rong Deng^1,2^, Shi-Juan Mai^1,2^, X.F. Steven Zheng^5^, Yibin Kang^6^, Hui-Yun Wang^1,2^**

**1. Supplemental Figures**

**2. Supplemental Table**

**
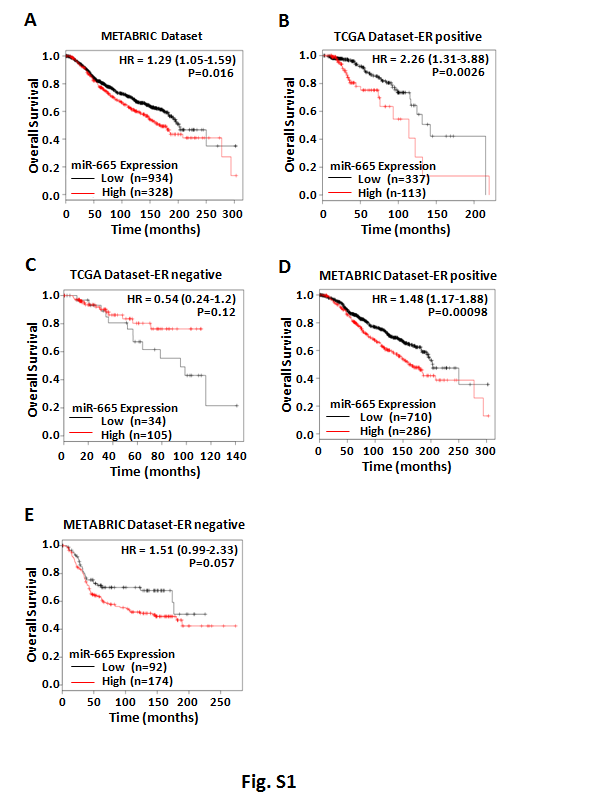
**

**Supplemental Figure S1. MiR-665 predicted poor prognosis of BC patients.** **(A)** BC samples obtained from the METABRIC showed in the Kaplan-Meier plotter database suggested that high expression levels of miR-665 was significantly associated with poorer overall survival of BC patients. **(B-E)** BC samples obtained from TCGA database (B-C) and the METABRIC database (D-E) showed in the Kaplan-Meier plotter database indicated that BC patients who expressed high levels of miR-665 in combination with positive ER were apt to live a shorter life than with negative ER of BC patients significantly (*P*<0.01, independent Student’s t-test).

**
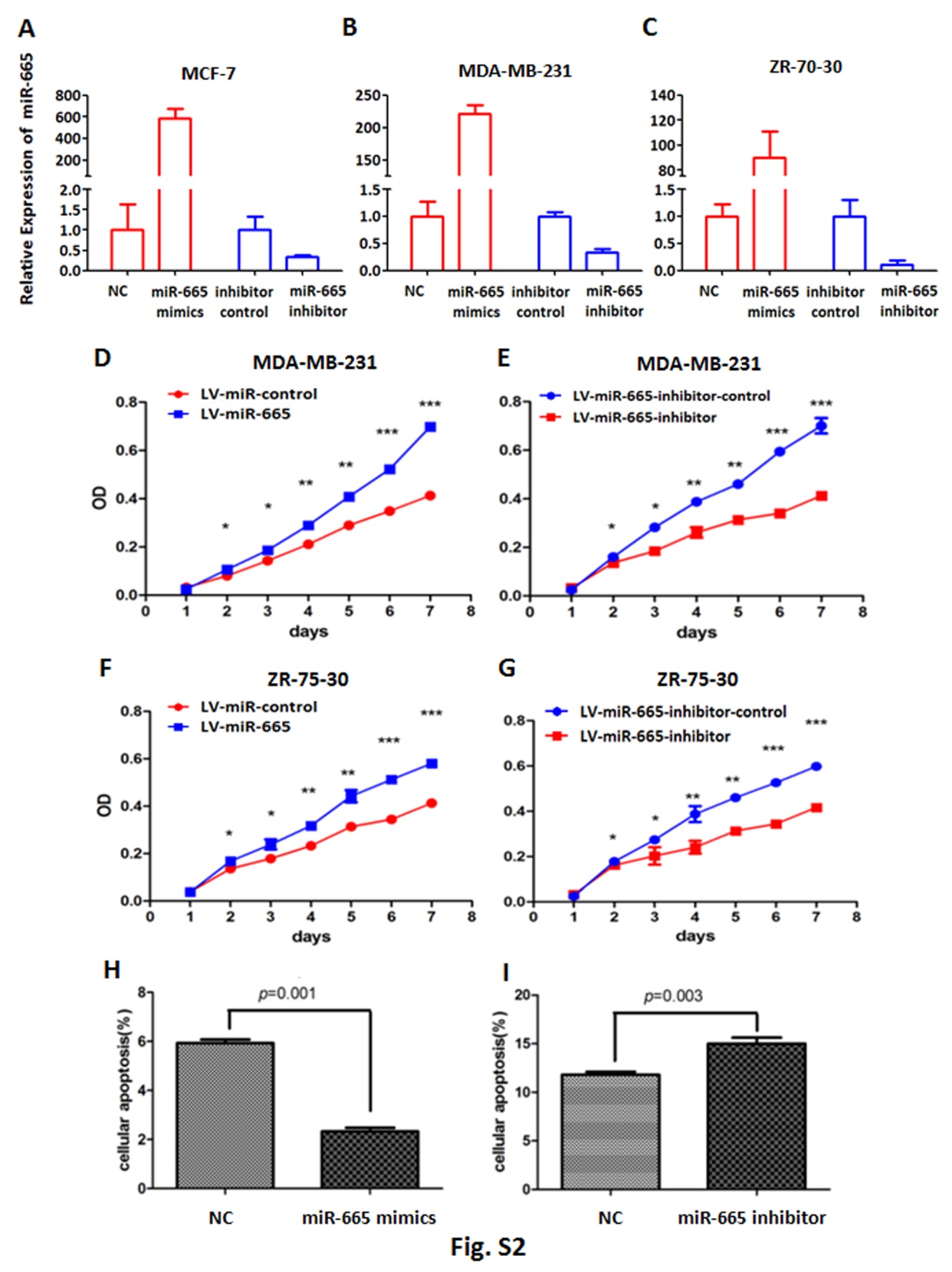
**

**Supplemental Figure S2. MiR-665 promoted BC proliferation and inhibited BC apoptosis. (A-C)** In the histogram, mRNA levels of miR-665 was upregulated induced by treatment of miR-665 (50 nM) mimics and downregulated induced by treatment of miR-665 inhibitor (50 nM) compared with the corresponding control groups in MCF-7 (A), MDA-MB-231 (B) and ZR-75-30 cells (C). **(D-G)** Overexpression of miR-665 significantly promoted cell viability in MDA-MB-231 (D) and ZR-75-30 cells (F) while knockdown of miR-665 significantly inhibited cell viability in MDA-MB-231 (E) and ZR-75-30 cells (G). **(H-I)** In the flow cytometry assay, the MCF-7 cells transfected with miR-665 mimics had the decreased apoptotic cells (H) while the MCF-7 cells transfected with miR-665 inhibitors had the increased apoptotic cells (I) compared with their control cells, respectively.

**
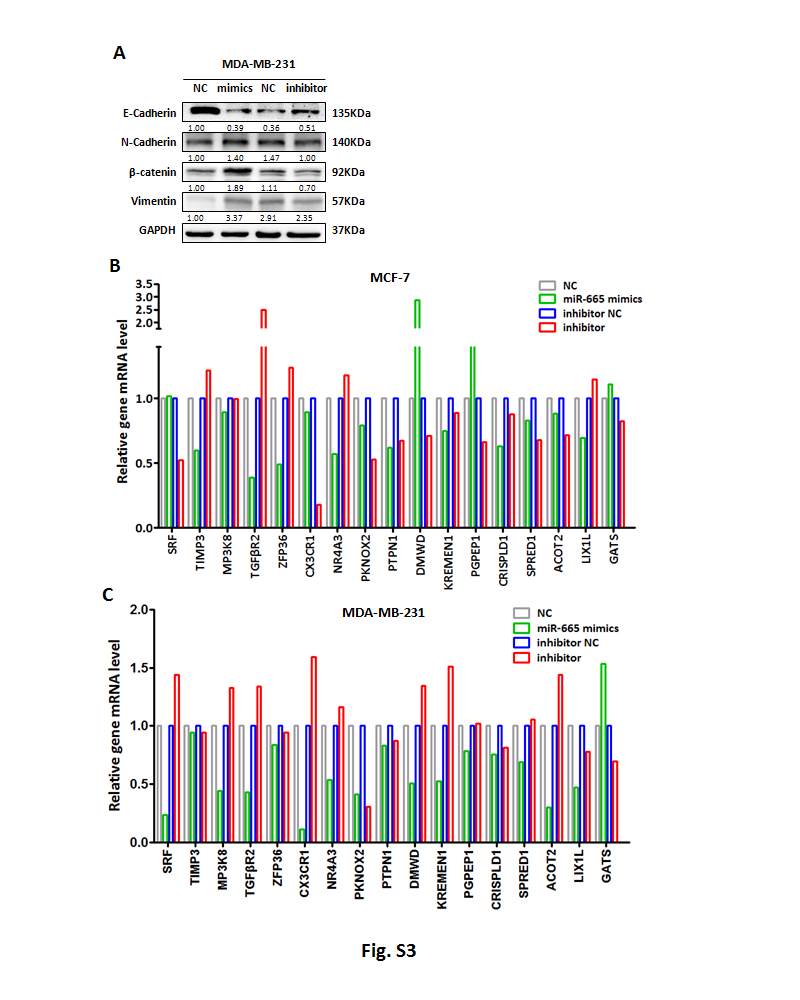
**

**Supplemental Figure S3.** **NR4A3 and TGFBR2 were direct target of miR-665. (A)** In western blotting analysis, overexpression of miR-665 decreased the level of epithelial maker (E-cadherin) and increased the levels of mesenchymal markers (N-cadherin, Vimentin and β-catenin) in MDA-MB-231 cells and the reverse results were observed in the cells with miR-665 downregulation. Numbers show the ratio of phosphor-protein/total protein (arbitrary unit). **(B-C)** In the histogram, mRNA expressions examined by qRT-PCR of 17 possible miR-665 target genes were compared after the treatment of miR-665 mimics or miR-665 inhibitor compared with the control group in MCF-7 cells (B) and MDA-MB-231 cells (C).

**
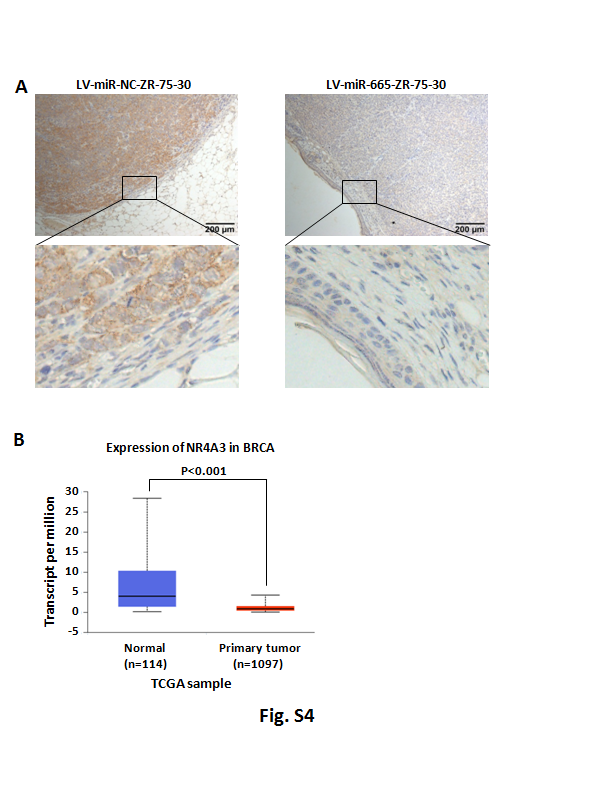
**

**Supplemental Figure S4.** **Expression of NR4A3 was downregulated in nude mice and patients. (A)** In IHC analysis, expression of NR4A3 was obviously downregulated in tumors ( right) formed in nude mice which were injected with LV-miR-665-ZR-75-30 cells compared with tumors ( left) formed in nude mice of which were injected with LV-miR-NC-ZR-75-30 cells. NR4A3 was downregulated in tumors compared with that in the control tumors in nude mice generated from LV-miR-control-ZR-70-30 cells. **(B)** In the histogram, samples from TCGA database showed that NR4A3 expressed lower in BC tissues (n=1097) rather than normal breast tissues (n=114) (*P*<0.001, independent Student’s t-test).

**Supplemental Table S1 Primers used in this study**

| Primers for q-PCR | Sequences |
| --- | --- |
| GAPDH Forward | CTCCTCCTGTTCGACAGTCAGC |
| GAPDH Reverse | CCCAATACGACCAAATCCGTT |
| NR4A3 Forward | TGCGTCCAAGCCCAATATAGC |
| NR4A3 Reverse | GGTGTATTCCGAGCTGTATGTCT |
| TGFβR2 Forward | ATGGAAGAGTGCAACGATTACAT |
| TGFβR2 Reverse | TGGCGCAGTTGTCACTGAAAT |
| miR-665 mimics Forward | ACCAGGAGGCUGAGGCCCCU |
| miR-665 mimics Reverse | GGGCCUCAGCCUCCUGGUUU |
| miR-665 mimics NC Forward | UUCUCCGAACGUGUCACGUTT |
| miR-665 mimics NC Reverse | ACGUGACACGUUCGGAGAATT |
| miR-665 inhibitor | AGGGGCCUCAGCCUCCUGGU |
| miR-665 inhibitor NC | CAGUACUUUUGUGUAGUACAA |
| U6 Forward | CTCGCTTCGGCAGCACA |
| U6 Reverse | AACGCTTCACGAATTTGCGT |
| NR4A3 siRNA: |  |
| NR4A3- si#1 Forward | GCCUUCCUGCGUGUACCAATT |
| NR4A3- si#1 Reverse | UUGGUACACGCAGGAAGGCTT |
| NR4A3- si#2 Forward | GCUGUUUGUCCUCAGACUUTT |
| NR4A3- si#2 Reverse | AAGUCUGAGGACAAACAGCTT |
| NR4A3- si#3 Forward | GCUCUUCCUGGACACCCUATT |
| NR4A3- si#3 Reverse | UAGGGUGUCCAGGAAGAGCTT |
| NR4A3- si#4 Forward | GCUUCCCGCUCUUCCACUUTT |
| NR4A3- si#4 Reverse | AAGUGGAAGAGCGGGAAGCTT |
| Control siRNA: |  |
| NR4A3- si#NC Forward | GAGGCGAAGGATGACAAAGGGA |
| NR4A3 -si#NC Reverse | GACAGATGTCCAGCCACAATTCT |
| TGFβR2 site1 Forward (PCR) | AGCACCTTCCATATGCCAATCGGGAGCACGCAC |
| TGFβR2 site1 Reverse (PCR) | GTGCGTGCTCCCGATTGGCATATGGAAGGTGCT |
| TGFβR2 site2 Forward | AATGGATAGATATATGCCAACTTACAAAACAGTT |
| TGFβR2 site2 Reverse | AACTGTTTTGTAAGTTGGCATATATCTATCCATT |
| miR-665 | TCTCCTCGAGGGGTCTCTGCCTCTACCCAGGACTCTTTCA TGACCAGGAGGCTGAGGCCC CTCACAGGCG GC |
| SRF Forward | GATCGGTATGGTGGTCGGTG |
| SRF Reverse | CAGTTTTCGGGTGGCAAAGG |
| TIMP3 Forward | TGTGCAACTTCGTGGAGAGG |
| TIMP3 Reverse | GAGCATGTCGGTCCAGAGAC |
| MP3K8 Forward | ATGGAGTACATGAGCACTGGA |
| MP3K8 Reverse | GCTGGCTCTTCACTTGCATAAAG |
| ZFP36 Forward | GACTGAGCTATGTCGGACCTT |
| ZFP36 Reverse | GAGTTCCGTCTTGTATTTGGGG |
| CX3CR1 Forward | CCTGGAGTATCTGAGCTTGCC |
| CX3CR1 Reverse | CTAGTCCTGTGCACCTACCTG |
| PKNOX2 Forward | GACGCTGCTGTTTGAGAAATG |
| PKNOX2 Reverse | ATCGCTGAAGAAGGGTTTGTG |
| PTPN1 Forward | TCCCTTTGACCATAGTCGGAT |
| PTPN1 Reverse | GTGACCGCATGTGTTAGGCA |
| DMWD Forward | CCACGATTTCAACCAGTTCACT |
| DMWD Reverse | GGTCTTGTCGATCAACCGCTC |
| KREMEN1 Forward | CCCGAGTGTTTCACAGCCAAT |
| KREMEN1 Reverse | GGATGCTGGAAAGTCTCGTTC |
| PGPEP1 Forward | CTGTGTGCAAGCGAGTCAC |
| PGPEP1 Reverse | GGTGTAGTAGGTAAAGTCGCAGA |
| CRISPLD2 Forward | GCCCAACGTCACTCTCTTAGA |
| CRISPLD2 Reverse | GTTGTGCAGCATGAGGATCTC |
| SPRED1 Forward | CAGCCAGGCTTGGACATTCA |
| SPRED1 Reverse | TGGGACTTTAGGCTTCCACAT |
| ACOT2 Forward | CGTCCCGGCTGTACCAATG |
| ACOT2 Reverse | GGAACCCTAATGATCTGACCAAC |
| LIX1L Forward | GCCCTCCTTATGTCTGCTATGT |
| LIX1L Reverse | ACTCATCAGTGATTCTTCGGGA |
| GATS Forward | AAACAGGTGTGGGGACGAG |
| GATS Reverse | TTCTGCTCTGGGTAACCGC |
